# A scoping review of risk behaviour interventions in young men

**DOI:** 10.1186/1471-2458-14-957

**Published:** 2014-09-16

**Authors:** Lee M Ashton, Melinda J Hutchesson, Megan E Rollo, Philip J Morgan, Clare E Collins

**Affiliations:** School of Health Sciences, Faculty of Health and Medicine, Priority Research Centre for Physical Activity and Nutrition, University of Newcastle, Callaghan Campus, Newcastle, Australia; School of Education, Faculty of Education and Arts, Priority Research Centre for Physical Activity and Nutrition, University of Newcastle, Callaghan Campus, Newcastle, Australia

**Keywords:** Scoping review, Young men, Risk behaviours, Health behaviour, Risk reduction behaviour, Intervention

## Abstract

**Background:**

Young adult males commonly engage in risky behaviours placing them at risk of acute and chronic health conditions. The purpose of this scoping review was to provide an overview of existing literature, describing the interventions targeting risk behaviours in young adult males.

**Methods:**

A search of seven electronic databases, grey literature and relevant journals reported in English language until May 2013 was conducted. All interventions that promoted healthy behaviours or reductions in risky behaviours to treat or prevent an associated health issue(s) in young adult males (17-35 years) in upper-middle and high-income countries were included. For inclusion the appropriate age range had to be reported and the sample had to be young adult male participants only or the outcomes reported with stratification by age and/or sex to include young adult males. Risk behaviours included: physical inactivity, poor diet, alcohol use, tobacco smoking, recreational drug use, unsafe sexual behaviours, tanning/sun exposure, violence, unsafe vehicle driving, gambling and self-harm.

**Results:**

The search strategy identified 16,739 unique citations and the full-text of 1149 studies were retrieved and screened with 100 included studies focussed on: physical inactivity (27%), alcohol use (25%), unsafe sexual behaviour (21%), poor diet (5%), unsafe vehicle driving (5%), tobacco smoking (4%), recreational drug use (2%), and tanning/sun exposure (1%) with no relevant studies targeting violence, gambling or self-harm. Also 10% of the studies targeted multiple risk behaviours. The most common study design was randomized controlled trials (62%). Face-to-face was the most common form of intervention delivery (71%) and the majority were conducted in university/college settings (46%). There were 46 studies (46%) that included young adult male participants only, the remaining studies reported outcomes stratified by age and/or sex.

**Conclusion:**

Risk behaviours in young men have been targeted to some extent, but the amount of research varies across risk behaviours. There is a need for more targeted and tailored interventions that seek to promote healthy behaviours or decrease risky behaviours in young men.

**Electronic supplementary material:**

The online version of this article (doi:10.1186/1471-2458-14-957) contains supplementary material, which is available to authorized users.

## Background

Risk behaviours are defined as actions that potentially have adverse effects on health and increase the likelihood of developing a specific disease [1]. Examples include: physical inactivity, unhealthy dietary habits, unsafe sexual behaviours, tobacco smoking, alcohol use, recreational drug use, unsafe vehicle driving, violence [2], self-harm [3], gambling [4] and tanning/sun exposure [5]. Risk behaviours contribute immensely to morbidity and mortality in men worldwide with tobacco use, physical inactivity and low fruit and vegetable intake responsible for 11%, 5% and 3% of total mortality globally, respectively [6]. The leading risks contributing to the global burden of disease in males include alcohol use, tobacco smoking and unsafe sex with each contributing 7%, 5% and 4% to total Disability-Adjusted Life Years (DALYs) [6]. Despite this, interventions targeting specific risk behaviours have not been consistently efficacious in achieving positive health behaviour change [7]. For instance, a meta-synthesis of 62 meta-analyses established that interventions targeting several behaviours (diet, physical activity, sexual behaviours and addictive behaviours) at individual-level, group-level and population-level had small to medium effect sizes (range = 0.08-0.45, mean effect size = 0.21) with the efficacy varying across the different behaviour domains [7].

A key concern is the young age at which individuals commence risky behaviours. Recent estimates indicate that one-third of the total disease burden in adults is due to risk behaviours that start in youth [2]. Engaging in risk behaviours at an early age is related to detrimental impacts on health including obesity, sexually transmitted diseases and specific cancers [8–10]. Research in the USA has shown that mortality from risk behaviours (suicide, motor vehicle accidents and unintentional injuries) in young adult males is 137 per 100,000 deaths, almost three times greater than for young females [11]. In addition the prevalence of obesity in young men is a concern; in the USA 29% (aged 20-39 yr) are classed as obese [12]. In Australia 15% (aged 18-24 yr) and 23% (aged 25-34 yr) are obese [13] and in England 12% (aged 16-24 yr) and 17% (aged 25-34 yr) are obese [14]. Moreover, contributors to obesity include physical inactivity, with approximately 20% of young men (aged 18-39 yr) failing to meet the national targets [15]. Poor diet quality, characterised by high intakes of energy dense foods and inadequate intakes of key food groups such as fruit, vegetables, wholegrain, legumes and nuts also contribute to the high prevalence of obesity [10]. In the USA 89% of young men (aged 19-30 yr) are failing to consume minimum recommended intakes for total fruit and 95% for total vegetables [16]. Similar inadequacies are apparent in Australia with 97% of young men (18-24 yr) failing to meet national recommendations for fruit and vegetables [13].

Tobacco smoking is the leading risk factor for global mortality in men [6]; 22% of young Australian men (aged 18-24 yr) [13] and 20% of young men from the USA [17] are classed as current daily tobacco smokers. Alcohol misuse is the third leading risk factor for global mortality in males (6.2% of deaths) [6]. Half (52.3%) of USA young adult men exceed daily maximum recommended alcohol intakes [18]. Alcohol consumption may be associated with other risky behaviours as it reduces inhibitions thereby increasing the likelihood of other behaviours including tobacco smoking, injuries, driving under the influence, violence and unsafe sex [2, 9, 19, 20].

Gambling behaviours are also a concern in young adults with almost 10% of college students in the USA and Canada qualifying as problem gamblers and 6% as pathological gamblers [4]. Sun exposure is another risk behaviour of concern in USA young men as they account for more than 60% of melanoma deaths [5] which is the most common form of cancer in young adults [21].

In young adult men social influences such as peer pressure [22], and environmental changes including leaving home, starting tertiary education, cohabitation and beginning employment [23] may provide some explanation for the widespread prevalence of risk behaviours in this population. Early work linking masculinity with men’s health often associated dominant forms of masculinity (i.e. Hegemonic masculinity) with poorer health behaviours [24–26]. Despite this, hegemonic masculinity is often encouraged in society amongst young men e.g. "take it like a man" [27] and in young men physical risk is often promoted and celebrated [28]. Young men who align to hegemonic masculinity may feel they are ‘invincible’ and hence take risks without fear of consequences [29], which supports the notion that hegemonic masculinities are strong predictors of risk behaviours [30]. This is not to say this is the case for all young men but may provide justification for the undertaking of risk behaviours in particular sub-groups of young men.

We need greater understanding of how sex and gender influences risky behaviours and health practices. Health research often fails to consider sex and gender in design, data collection and/or analysis; instead a generic ‘one size fits all’ approach is often adopted [31]. There is a need for more targeted interventions (i.e. those intended to reach a specific population subgroup [32]) and tailored interventions (those intended to reach one specific person, based on known differences that exist between individuals [32]) in young men because men’s needs and behaviours differ across the lifespan [33] and are different to women [34]. By utilizing a targeted and/or tailored approach the unique needs and interests of young men can be accounted for and help achieve the desired behaviour change [35]. According to the ‘elaboration likelihood model’ [36] more people are likely to process and retain information if they perceive it to be personally relevant, thus increasing the likelihood of attitude change [37]. In interventions directed at overweight/obesity [38], physical activity [39] and smoking cessation [40] research has confirmed the need for targeted and/or tailored approaches to engage men. Research has also drawn attention to the evidence gap for young men by emphasizing the need for targeted and/or tailored interventions in obesity treatment [41] and prevention [42], mental health [43] unsafe sexual behaviour and HIV prevention [44] and alcohol use [45]. Therefore, the purpose of this scoping review is to provide a broad overview of interventions that have either examined risk behaviours exclusively in young adult male population groups and those that have reported outcomes stratified by age and/or sex to identify outcomes in young adult males. The scoping review design will allow examination of the extent, range and nature of research activity, will help to summarise and disseminate research findings and can also serve as a foundation for more detailed systematic reviews [46]. Any identified gaps in the literature can guide future research of risk behaviour interventions in young men.

## Methods

The methodological framework as proposed by Arksey and O’Malley [46] was used to guide the scoping review. This framework provides a robust foundation for scoping study methodology and guidance to researchers undertaking scoping reviews [46]. The components of the framework used to guide this scoping review include: identifying the research question, searching for relevant studies, selecting studies, charting the data and collating, summarizing, and reporting the results.

### Identification of the research question to be addressed

The research question was defined using the PICOS (Population-Intervention-Comparison-Outcome-Study design) format. The question was designed to be broad and comprehensive to capture the full breadth of the literature and is a key aspect in scoping the field [46].

#### Participants

A population of young adult males (aged 17-35 years) was required for inclusion, with the appropriate age range specified within the article. Both the National Institute of Health [47] and the European commission of Men’s health [29] have used 18-35 years to define young adults. The inclusion criteria for participants was shaped around this definition, but the rationale for including age 17 was due to unsafe vehicle driving, as this is the age a driving license can be obtained in most countries. Studies were excluded if all participants had a major chronic medical condition(s). Research from lower income countries and low-to-middle income countries were excluded because of the large heterogeneity between low-income/low-to-middle income and upper-middle/high-income countries (e.g. the intervention characteristics of a HIV prevention intervention in a low-income/low-to-middle income country is unlikely to be translatable to an upper-middle/high-income country). The income group of each country was determined from the World Bank Group website [48]. Studies containing both males and females were included if results had been stratified by young adult males. Similarly studies containing old and young adults were only included if results had been stratified by young adult males. Stratified results specifically for young adult males must be reported in the Results section, as opposed to including ‘sex’ in regression models.

#### Interventions

Interventions designed to promote healthy behaviour (specific to the behaviours outlined in Table 1) or decrease risky behaviour to treat or prevent the associated health problem(s) (Table 1) in young adult males were accepted for inclusion. Any interventions targeting the risk behaviours as outlined in the World Health Organization were accepted for inclusion [2]. Gambling, tanning/sun exposure and self-harm were also included due to their high prevalence rates in young men [4, 5, 11, 21, 49]. In addition, interventions targeting multiple risk behaviours were included.

**Table 1:** **Included outcomes of interest**

Risk behaviour	Associated health outcomes
**Physical inactivity**	Obesity, diabetes, hypertension, hyperglycaemia, cancer, CVD, mental health disorders (i.e. depression), quality of life.
**Poor diet**	Obesity, diabetes, hypercholesterolemia, hypertension, hyperglycaemia, CVD, quality of life.
**Alcohol use**	Actual injury, alcohol dependence, obesity, diabetes, mental health disorders (i.e. depression), cancer, liver disease, CVD, quality of life.
**Tobacco smoking**	Respiratory/lung diseases, cancer, CVD, hypertension, hyperglycaemia, quality of life.
**Recreational drug use**	Mental health disorders (i.e. depression, anxiety), HIV, hepatitis.
**Sexual behaviours**	HIV, STD’s.
**Violence**	Actual injury, mental health disorders (i.e ADHD, schizophrenia, depression etc)
**Unsafe vehicle driving**	Actual injury, mental health disorders (i.e ADHD, bi-polar, schizophrenia, anxiety etc)
**Gambling**	Mental health disorders (i.e. depression), quality of life.
**Tanning/ sun exposure**	Skin cancer
**Self-harm**	Mental health disorders (i.e. depression), quality of life.

#### Outcomes of interest

To be included, the intervention had to aim to change one of the risk behaviours and have measured as an outcome change in that risk behaviour and/or the associated health outcome (Table 1).

#### Study design

The following experimental study designs were included in the review: randomized controlled trials, cluster randomized controlled trials, cluster controlled studies, non-randomized controlled trials (including quasi-experimental or controlled clinical trial), case series (including pre-test/ post-test design or before-after studies with no control) and interrupted time series. Systematic reviews and meta-analyses were included in the search strategy so that the reference lists of relevant reviews could be searched for additional relevant articles.

### Identification of studies relevant to the research question

#### Search strategy

The databases searched were: MEDLINE (Ovid) MEDLINE in process (Ovid), EMBASE (Ovid), PsycINFO (Ovid), Science Citation Index (WoS), Cinahl (EbscoHost) and Cochrane Library (Wiley). To locate relevant grey literature we used the ‘Grey Matters search tool’, ‘The grey literature report’, ‘Open Grey’ and the clinical trials registry (clinicaltrials.gov). All sources were searched from the date of inception to May 2013. Restrictions were applied to include only English language articles and human participants. The search strategy was developed by the research team with consultation from a medical librarian. The search consisted of focussed ‘text word’ searches and subject heading searches (MeSH). Our search strategy from the electronic databases can be found in Additional file 1. In addition all of the conference proceeding abstracts picked up in the bibliographic databases were assessed for inclusion and a reference list search of included studies, relevant systematic reviews and meta-analyses were conducted. We also searched the following key male health journals: *International Journal of Men’s Health, Journal of Men’s Health and American Journal of Men’s Health* from the first published article to the most recent published articles (Earliest: February 2004 and latest: November 2013).

### Selection of studies to include in the review

After removal of duplicates one reviewer conducted a screening review of the titles and excluded those which did not meet the inclusion criteria. One reviewer screened the abstracts and divided into three categories (Category 1: Relevant, Category 2: Not relevant, Category 3: Potentially relevant but failed to specify if results were stratified by age and/or sex). For those in category 3 we randomly selected and retrieved 10% (n = 104) of the full text articles to identify if results have been stratified by age and/or sex. None of these articles were deemed relevant after full text screening so the remaining articles in category 3 were excluded without further review. A second reviewer independently reviewed 10% of all abstracts (n = 1232) and an agreement of 97.6% was achieved. A third reviewer was consulted to resolve any disagreements. The full text articles of studies that were believed to satisfy the inclusion criteria (Category 1) were retrieved and a final decision on inclusion was made by one reviewer with a second reviewer consulted for any uncertainties. A third reviewer independently reviewed 10% of all full text papers (n = 115) and an agreement of 100% was achieved.

### Charting of information and data within the included studies

Data were extracted from included studies by one reviewer. For each study this included: author, year of publication, study design, risk behaviour targeted, associated health outcome targeted, target population group (e.g. young adult male participants only or results reported by strata for young adult males), sex and profession of intervention facilitator, sample age range, sample size, sample recruitment setting (e.g. university/college, community, hospital etc.), country of implementation, number of study arms, intervention strategy/study aim, theoretical model used to develop intervention, mode/delivery of intervention and intervention length and follow-up.

### Collating, summarising and reporting results of the review

As is convention in scoping reviews, a numerical analysis was undertaken to elucidate the extent and nature of the studies [50].

## Results

### Citation retrieval

**Figure 1:**
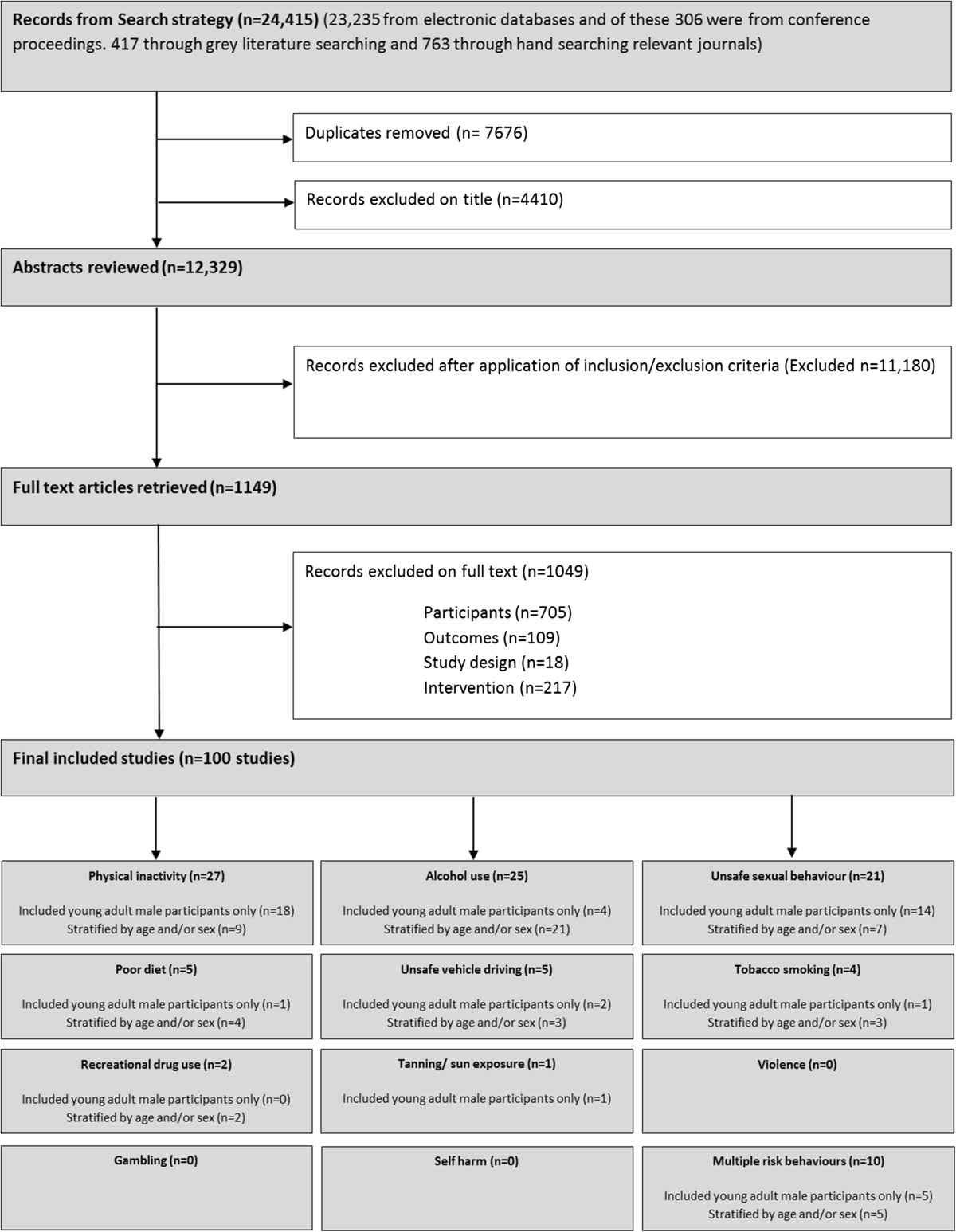
**Flow chart of studies through the scoping review process.**

The search strategy identified 24,415 citations (Figure 1). After removal of duplicates 16,739 citations were screened based on title and subsequently the abstracts of 12,329 citations were examined. Following abstract review 1149 remained for full text screening, of which 1049 articles were excluded. This gave 100 studies included in the scoping review.

The 100 included studies appeared in 70 journals and the year of publication ranged from 1972-2013, with 72 studies (72%) published from 2003 onwards. In addition one study was a grey literature paper, identified from the clinical trials registry (clinicaltrials.gov) [51]. The number of studies targeting each risk behaviour included 27 (27%) for physical inactivity [52–78], 25 (25%) for alcohol use [65–89], 21 (21%) for unsafe sexual behaviour [51, 79–98], 5 (5%) for poor diet [99–103], 5 (5%) for unsafe vehicle driving [104–108], 4 (4%) for tobacco smoking [109–112], 2 (2%) for recreational drug use [113, 114] and 1 (1%) for tanning/sun exposure [115]. There were 10 studies (10%) that assessed multiple risk behaviours [116–125] (of these 3 studies targeted poor diet and physical inactivity [120, 122, 123], 2 studies targeted alcohol use and recreational drug use [117, 121], 1 study targeted alcohol use and unsafe sexual behaviour [118], 1 study targeted poor diet, physical inactivity and tobacco smoking [116], 1 study targeted tobacco smoking, alcohol use and recreational drug use [119], 1 study targeted alcohol use and physical inactivity [125] and 1 study targeted unsafe vehicle driving and alcohol use [124]). No studies targeted the prevention or treatment of violence, gambling or self-harm.

Of the 100 included studies, 46 included young male participants only with 18 (18% of total studies and 39.1% of young adult male only studies) [52–54, 57, 58, 60, 61, 64–73, 76] for physical inactivity, 14 (14% of total studies and 30.4% of young adult male only studies) for unsafe sexual behaviour [51, 80, 82, 85–91, 94, 96–98], 4 (4% of total studies and 8.7% of young adult male only studies) for alcohol use [126–129], 2 (2% of total studies and 4.3% of young adult male only studies) for unsafe vehicle driving [104, 105] 1 (1% of total studies and 2.2% of young adult male only studies) for tobacco smoking [110], 1 (1% of total studies and 2.2% of young adult male only studies) for poor diet [103], and 1 (1% of total studies and 2.2% of young adult male only studies) for tanning/sun exposure [115]. Also 5 (5% of total studies and 10.9% of young adult male only studies) of the studies with only young adult male participants targeted multiple risk behaviours [116, 119, 122, 124, 125]. See Additional file 2 for detailed information on the included studies, such as: author(s), title, year, study design, country, target population group, age range and risk behaviour targeted.

### Intervention country and risk behaviours

The interventions were conducted across several continents, with most conducted in North America (n = 63, 63%) [45, 51, 57–59, 62, 63, 68, 73–75, 77, 78, 80, 82, 85–94, 96, 97, 99–101, 103, 106, 109, 111–113, 115, 117, 118, 121–123, 125, 126, 129–147] (the majority of these were conducted in the USA n = 61). The remaining interventions were carried out in Europe (n = 18, 18%) [52, 53, 55, 56, 60, 61, 70, 81, 95, 98, 102, 104, 110, 116, 119, 127, 128, 148], Australasia (n = 8, 8%) [54, 65, 66, 105, 108, 120, 124, 149], Asia (n = 6, 6%) [64, 67, 71, 72, 107, 114], South America (n = 3, 3%) [69, 79, 83], Africa (n = 1, 1%) [76] and one intervention (1%) was implemented in multiple countries across two continents (USA, Ecuador, Peru and Brazil) [84]. The risk behaviours that were targeted in various intervention countries, separated by studies with young adult male participants only and studies with outcomes stratified by age/sex to include young adult males are shown in Table 2. It is clear that the interventions targeting alcohol use were predominantly performed in the USA (84%) and a similar pattern occurred for unsafe sexual behaviour (66.7%). Although the USA was the leading intervention country in the other risk behaviours, the pattern tended to be less disproportionate.

**Table 2:** **Intervention country by risk behaviours**

Intervention country	Physical inactivity	Poor diet	Alcohol use	Tobacco smoking	Recreational drug use	Unsafe sexual behaviour	Violence	Unsafe vehicle driving	Gambling	Tanning/sun exposure	Self-harm	Multiple risk behaviours	Total
***Included young adult male participants only***													
**USA**	4	1	2	0	0	12	0	0	0	1	0	2^*,†^	22
**UK**	0	0	0	0	0	1	0	0	0	0	0	0	1
**Australia**	3	0	0	0	0	0	0	1	0	0	0	1^‡^	5
**Brazil**	1	0	0	0	0	0	0	0	0	0	0	0	1
**Turkey**	1	0	0	1	0	0	0	0	0	0	0	0	2
**Finland**	4	0	0	0	0	0	0	0	0	0	0	0	4
**Iran**	1	0	0	0	0	0	0	0	0	0	0	0	1
**Israel**	2	0	0	0	0	0	0	0	0	0	0	0	2
**South Africa**	1	0	0	0	0	0	0	0	0	0	0	0	1
**Thailand**	1	0	0	0	0	0	0	0	0	0	0	0	1
**France**	0	0	0	0	0	0	0	0	0	0	0	1^****^	1
**Switzerland**	0	0	2	0	0	0	0	0	0	0	0	1^§^	3
**Sweden**	0	0	0	0	0	0	0	1	0	0	0	0	1
**Canada**	0	0	0	0	0	1	0	0	0	0	0	0	1
**Total**	18	1	4	1	0	14	0	2	0	1	0	5	46
***Studies with outcomes stratified by age and/or sex to include young adult males***													
**USA**	7	3	19	3	1	2	0	1	0	0	0	3 ^**,***,**^	39
**Australia**	0	0	0	0	0	0	0	1	0	0	0	1^*^	2
**Brazil**	0	0	0	0	0	1	0	0	0	0	0	0	1
**Turkey**	2	0	0	0	0	0	0	0	0	0	0	0	2
**Israel**	0	0	0	0	0	0	0	1	0	0	0	0	1
**Mexico**	0	0	0	0	0	0	0	0	0	0	0	1^*^	1
**Denmark**	0	1	0	0	0	0	0	0	0	0	0	0	1
**Netherlands**	0	0	1	0	0	0	0	0	0	0	0	0	1
**New Zealand**	0	0	1	0	0	0	0	0	0	0	0	0	1
**China**	0	0	0	0	1	0	0	0	0	0	0	0	1
**Italy**	0	0	0	0	0	1	0	0	0	0	0	0	1
**Sweden**	0	0	0	0	0	1	0	0	0	0	0	0	1
**Peru**	0	0	0	0	0	1	0	0	0	0	0	0	1
**International (multiple countries)** ^**§§**^	0	0	0	0	0	1	0	0	0	0	0	0	1
**Total**	9	4	21	3	2	7	0	3	0	0	0	5	54
**Overall total**	27	5	25	4	2	21	0	5	0	1	0	10	100

^*^ Poor diet and Physical inactivity.

^**^ Alcohol use and Recreational drug use.

^***^ Alcohol use and Unsafe sexual behaviour.

^****^ Poor diet, physical inactivity and tobacco smoking.

^†^Alcohol use and physical inactivity.

^‡^ Unsafe vehicle driving and alcohol use.

^§^Tobacco smoking, alcohol use and recreational drug use.

^§§^ Countries included: USA. Ecuador, Peru and Brazil.

### Study designs and risk behaviours

Of the 100 included studies there were 62 (62%) randomized controlled trials [51–57, 59, 62–73, 75, 77, 78, 82, 84–86, 90, 92, 93, 96, 103, 105, 108, 109, 112, 113, 116, 118–120, 123–125, 127–131, 133–135, 138–140, 143–149], 16 (16%) case series [57, 60, 61, 74, 76, 80, 91, 94, 99–101, 110, 115, 117, 121, 136], 9 (9%) non-randomized controlled trials [87, 95, 98, 102, 104, 107, 122, 137, 141], 7 (7%) cluster randomized controlled trials [79, 81, 83, 111, 126, 132, 142], 4 (4%) cluster controlled studies [45, 88, 97, 114] and 2 (2%) interrupted time series [89, 106]. The number of papers for each study design and reported risk behaviour is provided in Table 3. The randomized controlled design had the greatest percentage use in the physical inactivity interventions (81.5%), whilst the unsafe sexual behaviour interventions had the greatest diversity in study design.

**Table 3:** **Study design by risk behaviours**

Study design	Physical inactivity	Poor diet	Alcohol use	Tobacco smoking	Recreational drug use	Unsafe sexual behaviour	Violence	Unsafe vehicle driving	Gambling	Tanning/sun exposure	Self-harm	Multiple risk behaviours	Total
***Included young adult male participants only***													
**RCT**	14	1	3	0	0	6	0	1	0	0	0	4^****, §, †, ‡^	29
**Non- RCT**	0	0	0	0	0	2	0	1	0	0	0	1^*^	4
**Case series**	4	0	0	1	0	3	0	0	0	1	0	0	9
**Cluster RCT**	0	0	1	0	0	0	0	0	0	0	0	0	1
**Cluster controlled**	0	0	0	0	0	2	0	0	0	0	0	0	2
**Interrupted time series**	0	0	0	0	0	1	0	0	0	0	0	0	1
**Total**	18	1	4	1	0	14	0	2	0	1	0	5	46
***Studies with outcomes stratified by age and/or sex to include young adult males***													
**RCT**	8	0	15	2	1	3	0	1	0	0	0	3^***, *,*^	33
**Non- RCT**	0	1	2	0	0	1	0	1	0	0	0	0	5
**Case series**	1	3	1	0	0	017	0	0	0	0	0	2^**, **^	7
**Cluster RCT**	0	0	2	1	0	3	0	0	0	0	0	0	6
**Cluster controlled**	0	0	1	0	1	0	0	0	0	0	0	0	2
**Interrupted time series**	0	0	0	0	0	0	0	1	0	0	0	0	1
**Total**	9	4	21	3	2	7	0	3	0	0	0	5	54
**Overall total**	27	5	25	4	2	21	0	5	0	1	0	10	100

^*^ Poor diet and Physical inactivity.

^**^ Alcohol use and Recreational drug use.

^***^ Alcohol use and Unsafe sexual behaviour.

^****^ Poor diet, physical inactivity and tobacco smoking.

^†^Alcohol use and physical inactivity.

^‡^ Unsafe vehicle driving and alcohol use.

^§^Tobacco smoking, alcohol use and recreational drug use.

### Intervention setting and risk behaviours

Interventions were implemented across a wide variety of settings with the most common being university/college settings (n = 46, 46%) [53–59, 62–66, 68, 70, 73, 75, 78, 93, 95, 96, 98–101, 103, 105, 108, 109, 112, 115, 117, 118, 121, 123–126, 129, 130, 134, 136, 137, 141, 144, 146, 147]. Other prevalent settings included military/army (n = 11, 11%) [52, 60, 61, 69, 71, 72, 104, 110, 119, 127, 128] and internet (n = 8, 8%) [45, 85, 92, 113, 132, 139, 148, 149]. Five studies (5%) were delivered in multiple settings [83, 111, 120, 122, 145]. The risk behaviours across the different settings are summarised in Table 4. The university/college setting was the most prevalent in the physical inactivity interventions (63%). This was also the leading setting (or equal leading) for seven out of the eight risk behaviours that were intervened upon and amongst the studies targeting multiple risk behaviours. All of the studies that utilized a military/army setting included young adult male participants only.

**Table 4:** **Intervention setting by risk behaviours**

Intervention setting	Physical inactivity	Poor diet	Alcohol use	Tobacco smoking	Recreational drug use	Unsafe sexual behaviour	Violence	Unsafe vehicle driving	Gambling	Tanning/sun exposure	Self-harm	Multiple risk behaviours	Total
***Included young adult male participants only***													
**University/ college**	10	1	2	0	0	2	0	1	0	1	0	2 ^†, ‡^	19
**Community**	0	0	0	0	0	4	0	0	0	0	0	0	4
**Primary- care setting**	0	0	0	0	0	4	0	0	0	0	0	1^****^	5
**Military/army**	6	0	2	1	0	0	0	1	0	0	0	1 ^§^	11
**Prison**	0	0	0	0	0	1	0	0	0	0	0	0	1
**Exercise laboratory**	2	0	0	0	0	0	0	0	0	0	0	0	2
**Internet**	0	0	0	0	0	1	0	0	0	0	0	0	1
**Home**	0	0	0	0	0	1	0	0	0	0	0	0	1
**Primary care + gym**	0	0	0	0	0	0	0	0	0	0	0	1^*^	1
**Not stated**	0	0	0	0	0	1	0	0	0	0	0	0	1
**Total**	18	1	4	1	0	14	0	2	0	1	0	5	46
***Studies with outcomes stratified by age and/or sex to include young adult males***													
**University/ college**	7	3	8	2	0	2	0	1	0	0	0	4*,^* **, ***,^	27
**School**	0	0	0	0	1	2	0	0	0	0	0	0	3
**Workplace**	1	0	0	0	0	0	0	0	0	0	0	0	1
**Community**	0	0	1	0	0	0	0	1	0	0	0	0	2
**Primary- care setting**	0	0	0	0	0	1	0	0	0	0	0	0	1
**Hospital**	0	0	2	0	0	0	0	0	0	0	0	0	2
**Exercise laboratory**	1	0	0	0	0	0	0	0	0	0	0	0	1
**Internet**	0	0	5	0	1	1	0	0	0	0	0	0	7
**Home**	0	1	4	0	0	0	0	0	0	0	0	0	5
**Car**	0	0	0	0	0	0	0	1	0	0	0	0	1
**Internet + home**	0	0	0	0	0	0	0	0	0	0	0	1*	1
**Internet + University/college**	0	0	1	1	0	0	0	0	0	0	0	0	2
**Community + Primary care**	0	0	0	0	0	1	0	0	0	0	0	0	1
**Total**	9	4	21	3	2	7	0	3	0	0	0	0	54
**Overall total**	27	5	25	4	2	21	0	5	0	1	0	10	100

^*^ Poor diet and Physical inactivity.

^**^ Alcohol use and Recreational drug use.

^***^ Alcohol use and Unsafe sexual behaviour.

^****^ Poor diet, physical inactivity and tobacco smoking.

^†^Alcohol use and physical inactivity.

^‡^ Unsafe vehicle driving and alcohol use.

^§^Tobacco smoking, alcohol use and recreational drug use.

### Intervention mode and risk behaviours

The included studies most commonly used ‘face-to-face’ delivery (n = 71, 71%) [51, 53–58, 60–82, 84, 86–91, 94, 96–105, 107–110, 112, 114, 117, 121, 123–130, 133–136, 144, 146, 147]. Other modes included online (n = 8, 8%) [45, 52, 85, 113, 132, 139, 148, 149], paper (n = 5, 5%) [137, 138, 140, 142, 143], TV/video/DVD (n = 2, 2%) [92, 93] and a social marketing campaign (n = 1, 1%) [106]. Many studies (n = 13, 13%) implemented multi-faceted intervention modes [59, 83, 95, 111, 115, 116, 118–120, 122, 131, 141, 145]. The delivery mode for each risk behaviour is shown in Table 5, with the face-to-face the predominant approach across all risk behaviours. This mode also featured most commonly (either alone or with other intervention mode(s)) in physical inactivity studies (92.6%). The use of technology based intervention modes (e.g. online or mobile phone) either alone or in combination with other mode(s) was less likely to be used in studies that included young adult male participants only.

**Table 5:** **Intervention mode by risk behaviour**

Intervention mode	Physical inactivity	Poor diet	Alcohol use	Tobacco smoking	Recreational drug use	Unsafe sexual behaviour	Violence	Unsafe vehicle driving	Gambling	Tanning/sun exposure	Self-harm	Multiple risk behaviour	Total
***Included young adult male participants only***													
**Online**	1	0	0	0	0	1	0	0	0	0	0	0	2
**Face to face**	17	1	4	1	0	13	0	2	0	1	0	2 ^†, ‡^	41
**Face-to-Face + Paper**	0	0	0	0	0	0	0	0	0	0	0	2^*, ****^	2
**Face-to Face + mobile phone**	0	0	0	0	0	0	0	0	0	0	0	1 ^§^	1
**Total**	18	1	4	1	0	14	0	2	0	1	0	5	46
***Studies with outcomes stratified by age and/or sex to include young adult males***													
**Online**	0	0	5	0	1	0	0	0	0	0	0	0	6
**Paper**	0	0	5	0	0	0	0	0	0	0	0	0	5
**Face to face**	8	4	7	2	1	3	0	2	0	0	0	3^*, **. **^	30
**TV/Video/DVD**	0	0	0	0	0	2	0	0	0	0	0	0	2
**Social marketing**	0	0	0	0	0	0	0	1	0	0	0	0	1
**Face-to-Face + Online + Mobile phone**	1	0	0	0	0	0	0	0	0	0	0	0	1
**Face-to-Face + Paper**	0	0	2	0	0	2	0	0	0	0	0	1^***^	5
**Online + Mobile phone**	0	0	0	0	0	0	0	0	0	0	0	1^*^	1
**Face-to Face + mobile phone**	0	0	1	0	0	0	0	0	0	0	0	0	1
**Face- to- Face + Online**	0	0	1	1	0	0	0	0	0	0	0	0	2
**Total**	9	4	21	3	2	7	0	3	0	0	0	5	54
**Overall total**	27	5	25	4	2	21	0	5	0	1	0	10	100

^*^ Poor diet and Physical inactivity.

^**^ Alcohol use and Recreational drug use.

^***^ Alcohol use and Unsafe sexual behaviour.

^****^ Poor diet, physical inactivity and tobacco smoking.

^†^Alcohol use and physical inactivity.

^‡^ Unsafe vehicle driving and alcohol use.

^§^Tobacco smoking, alcohol use and recreational drug use.

### Sex of the Intervention facilitator and risk behaviours

For those interventions delivered by individuals (n = 87), we examined who had delivered the intervention. Only 12 studies indicated the sex of the facilitator; of which 7 (7%) were delivered by males only [80, 82, 88, 89, 94, 98, 146], whilst 4 (4%) of the studies included personnel with mixed sexes [59, 87, 97, 144] and one study delivered by a female [129]. For a breakdown of delivery personnel sex by risk behaviour refer to Table 6. The use of a male facilitator was most common for unsafe sexual behaviour interventions (28.6%) and also more common in the studies with only young adult male participants (13%).

**Table 6:** **Sex of the intervention facilitator and risk behaviours**

Intervention delivered by	Physical inactivity	Poor diet	Alcohol use	Tobacco smoking	Recreational drug use	Unsafe sexual behaviour	Violence	Unsafe vehicle driving	Gambling	Tanning/sun exposure	Self-harm	Multiple risk behaviour	Total
***Included young adult male participants only***													
**Male only**	0	0	0	0	0	6	0	0	0	0	0	0	6
**Female only**	0	0	1	0	0	0	0	0	0	0	0	0	1
**Mixed**	0	0	0	0	0	2	0	0	0	0	0	0	2
**Not stated**	17	1	3	1	0	4	0	2	0	1	0	5^*, ****, †, ‡, §^	34
**N/A**	1	0	0	0	0	2	0	0	0	0	0	0	3
**Total**	18	1	4	1	0	14	0	2	0	1	0	5	46
***Studies with outcomes stratified by age and/or sex to include young adult males***													
**Male only**	0	0	1	0	0	0	0	0	0	0	0	0	1
**Female only**	0	0	0	0	0	0	0	0	0	0	0	0	0
**Mixed**	1	0	1	0	0	0	0	0	0	0	0	0	2
**Not stated**	8	4	13	3	1	6	0	2	0	0	0	4^*, **, **, ***^	41
**N/A**	0	0	6	0	1	1	0	1	0	0	0	1^*^	10
**Total**	9	4	21	3	2	7	0	3	0	0	0	5	54
**Overall total**	27	5	25	4	2	21	0	5	0	1	0	10	100

^*^ Poor diet and Physical inactivity.

^**^ Alcohol use and Recreational drug use.

^***^ Alcohol use and Unsafe sexual behaviour.

^****^ Poor diet, physical inactivity and tobacco smoking.

^†^Alcohol use and physical inactivity.

^‡^ Unsafe vehicle driving and alcohol use.

^§^Tobacco smoking, alcohol use and recreational drug use.

### Profession of the intervention facilitator and risk behaviours

Most often a member of the research team (n = 20, 20%) [54, 57, 58, 62, 63, 86, 94, 99–101, 104, 107, 109, 112, 134, 136–139, 143], personal trainer/training supervisors (n = 8, 8%) [56, 68, 71–74, 77, 78], educated supervisors/trained facilitators (n = 7, 7%) [60, 61, 90, 93, 97, 105, 145] and counsellors (n = 7, 7%) [59, 111, 117, 118, 127, 128, 131] were involved in the delivery. In addition, interventions regularly incorporated multiple professionals for delivery (n = 9, 9%) [75, 80, 81, 83, 87–89, 125, 144]. There were 20 (20%) studies that failed to state the profession [51, 53, 55, 64–67, 69, 70, 76, 79, 84, 102, 108, 114, 115, 124, 126, 130, 135] and 12 (12%) of the studies were self-directed [45, 52, 85, 91, 106, 113, 120, 132, 140, 142, 148, 149]. A summary of the risk behaviours and by whom the intervention was delivered by is available in Table 7. The profession of the facilitator tended to be diverse across the risk behaviours. Failing to report the profession of the facilitator was mainly done in the physical inactivity studies (33.3%).

**Table 7:** **Profession of the intervention facilitator and risk behaviours**

Intervention delivered by	Physical inactivity	Poor diet	Alcohol use	Tobacco smoking	Recreational drug use	Unsafe sexual behaviour	Violence	Unsafe vehicle driving	Gambling	Tanning/sun exposure	Self-harm	Multiple risk behaviour	Total
***Included young adult male participants only***													
**Doctor/physician/clinician**	0	0	0	0	0	0	0	0	0	0	0	1^****^	1
**Dietician**	0	1	0	0	0	0	0	0	0	0	0	1^*^	2
**Nurse**	0	0	0	1	0	0	0	0	0	0	0	0	1
**Member of research team**	3	0	0	0	0	2	0	1	0	0	0	0	6
**Personal trainer/ training supervisor**	4	0	0	0	0	0	0	0	0	0	0	0	4
**Educated supervisor/ Trained facilitators**	2	0	0	0	0	2	0	1	0	0	0	0	5
**Health educator/advisor**	0	0	0	0	0	1	0	0	0	0	0	0	1
**Peer educators**	0	0	0	0	0	2	0	0	0	0	0	0	2
**Counsellor**	0	0	2	0	0	0	0	0	0	0	0	0	2
**Psychologist**	0	0	1	0	0	0	0	0	0	0	0	1 ^§^	2
**Self-learn**	1	0	0	0	0	2	0	0	0	0	0	0	3
**Health educators + social worker + Doctor/physician/clinician**	0	0	0	0	0	1	0	0	0	0	0	0	1
**Peer educators + community advisory board**	0	0	0	0	0	2	0	0	0	0	0	0	2
**Doctor/physician/clinician + Nurses + Health educators**	0	0	0	0	0	1	0	0	0	0	0	0	1
**Trained facilitator + Nurse + Meditator**	0	0	0	0	0	0	0	0	0	0	0	1 ^†^	1
**Not stated**	8	0	1	0	0	1	0	0	0	1	0	1^‡^	12
**Total**	18	1	4	1	0	14	0	2	0	1	0	5	46
***Studies with outcomes stratified by age and/or sex to include young adult males***													
**Student**	0	0	3	0	0	0	0	0	0	0	0	1^**^	4
**Member of research team**	2	3	6	2	0	0	0	1	0	0	0	0	14
**Personal trainer/ training supervisor**	4	0	0	0	0	0	0	0	0	0	0	0	4
**Educated supervisor/ Trained facilitators**	0	0	1	0	0	1	0	0	0	0	0	0	2
**Peer educators**	0	0	0	0	0	1	0	0	0	0	0	0	1
**Counsellor**	1	0	1	1	0	0	0	0	0	0	0	2^**, ***^	5
**Psychologist**	0	0	0	0	0	0	0	0	0	0	0	1^*^	1
**Social Worker**	0	0	1	0	0	0	0	0	0	0	0	0	1
**Self-learn**	0	0	6	0	1	0	0	1	0	0	0	1^*^	9
**Member of research team + student**	1	0	0	0	0	0	0	0	0	0	0	0	1
**Psychologist + Student**	0	0	1	0	0	0	0	0	0	0	0	0	1
**Doctor/physician/clinician + Peer educators**	0	0	0	0	0	1	0	0	0	0	0	0	1
**Teachers + Peer educators**	0	0	0	0	0	1	0	0	0	0	0	0	1
**Not stated**	1	1	2	0	1	2	0	1	0	0	0	0	8
**N/A**	0	0	0	0	0	1	0	0	0	0	0	0	1
**Total**	9	4	21	3	2	7	0	3	0	0	0	5	54
**Overall total**	27	5	25	4	2	21	0	5	0	1	0	10	100

^*^ Poor diet and Physical inactivity.

^**^ Alcohol use and Recreational drug use.

^***^ Alcohol use and Unsafe sexual behaviour.

^****^ Poor diet, physical inactivity and tobacco smoking.

^†^Alcohol use and physical inactivity.

^‡^ Unsafe vehicle driving and alcohol use.

^§^Tobacco smoking, alcohol use and recreational drug use.

### Theoretical framework and risk behaviours

Only 19 studies (19%) reported utilising a theoretical framework to develop the intervention. The trans-theoretical model was most commonly used (n = 4, 4%) [52, 110, 112, 121], followed by social cognitive theory (n = 3, 3%) [81, 100, 101]. In addition several studies (n = 7, 7%) used multiple frameworks to help with development of the interventions [45, 59, 75, 90, 111, 114, 132]. A summary of the theoretical frameworks utilized, by risk behaviours, is summarised in Table 8. The most commonly used theoretical framework (either alone or with another framework/model) was the trans-theoretical model (10%). The risk behaviour that was most likely to report use of a theoretical framework was for poor diet (40%). Contrary to this, failure to report use of a theoretical framework was most common in unsafe vehicle driving interventions (100%).

**Table 8:** **Theoretical framework and risk behaviour**

Theoretical framework	Physical inactivity	Poor diet	Alcohol use	Tobacco smoking	Recreational drug use	Unsafe sexual behaviour	Violence	Unsafe vehicle driving	Gambling	Tanning/sun exposure	Self-harm	Multiple risk behaviour	Total
***Included young adult male participants only***													
**Trans-Theoretical Model**	1	0	0	1	0	0	0	0	0	0	0	0	2
**Integrated Model of Behaviour Theory**	0	0	0	0	0	1	0	0	0	0	0	0	1
**Social Cognitive Theory + Trans-Theoretical Model**	0	0	0	0	0	1	0	0	0	0	0	0	1
**Not stated**	17	1	4	0	0	12	0	2	0	1	0	5^*, ****, †, ‡, §^	42
**Total**	18	1	4	1	0	14	0	2	0	1	0	5	46
***Studies with outcomes stratified by age and/or sex to include young adult males***													
**Social Cognitive Theory**	0	2	0	0	0	1	0	0	0	0	0	0	3
**Trans-Theoretical Model**	0	0	0	1	0	0	0	0	0	0	0	1^**^	2
**Health Promotion Model**	0	0	0	0	0	0	0	0	0	0	0	1^*^	1
**Self-Regulation Theory**	0	0	1	0	0	0	0	0	0	0	0	0	1
**Theory of Cognitive conditioning**	0	0	0	1	0	0	0	0	0	0	0	0	1
**Process of Change Theory**	0	0	0	0	0	0	0	0	0	0	0	1^*^	1
**Social Cognitive Theory + Trans-Theoretical Model**	2	0	0	0	0	0	0	0	0	0	0	0	2
**Community Organization Model + Behaviour Change Model.**	0	0	0	0	1	0	0	0	0	0	0	0	1
**Trans-Theoretical Model + Health Belief Model**	0	0	0	1	0	0	0	0	0	0	0	0	1
**Trans-Theoretical Model + Health Belief Model + Theory of Planned Behaviour**	0	0	2	0	0	0	0	0	0	0	0	0	2
**Not stated**	7	2	18	0	1	6	0	3	0	0	0	2^**, ***^	39
**Total**	9	4	21	3	2	7	0	3	0	0	0	5	54
**Overall total**	27	5	25	4	2	21	0	5	0	1	0	10	100

^*^ Poor diet and Physical inactivity.

^**^ Alcohol use and Recreational drug use.

^***^ Alcohol use and Unsafe sexual behaviour.

^****^ Poor diet, physical inactivity and tobacco smoking.

^†^Alcohol use and physical inactivity.

^‡^ Unsafe vehicle driving and alcohol use.

^§^Tobacco smoking, alcohol use and recreational drug use.

## Discussion

The aim of this scoping review was to provide a broad overview of interventions that have targeted risk behaviours in young adult males aged 17 to 35 years. Although the search strategy identified over 16,000 articles, the final number of studies that only included young adult male participants was low (n = 46). The remaining studies (n = 54) stratified and reported outcomes by age and/or sex. The three risk behaviours most frequently targeted in interventions that have been conducted in young adult males to date were: physical inactivity (n = 27), alcohol use (n = 25), and unsafe sexual behaviours (n = 21). These three risk behaviours were also targeted most frequently in studies that included only young adult male participants (physical inactivity n = 18, unsafe sexual behaviour n = 14 and alcohol use n = 4). In comparison, there were smaller numbers of studies targeting poor diet, recreational drug use, tobacco smoking, unsafe vehicle driving and tanning/sun exposure, with no relevant studies targeting gambling, violence or self-harm.

The distribution of risk behaviours within the included interventions mostly corresponds with prevalence rates from USA-based studies. This and the heterogeneity in reporting makes it difficult to draw conclusions. Low fruit and vegetable intakes, alcohol use and obesity rates were the most prevalent risk behaviours in young men in the USA. Although this scoping review identified few interventions on poor diet in young men, there were greater numbers of interventions for physical inactivity. Given the high prevalence of alcohol use young men in the USA and that alcohol is the leading contributor to lost DALYs (7.4% of total DALYs) in males it is not surprising that a high proportion of interventions targeting alcohol use were identified in this review.

Few interventions included young adult male participants only (n = 46). Most studies targeted young adults in general (male and female) (n = 51) in the same intervention and then stratified results by sex. However, stratifying the results by sex and examining the sex comparisons for certain parameters can often obscure the within- and across sex variations which can limit the accuracy and applicability of results [31]. In addition the European commission for men’s health have highlighted that the biological and gender behaviour differences represent different needs and perceived barriers in terms of health promotion and intervention design [29]. Consequently, designing interventions for both sexes together is not likely to suit all characteristics of either sex, and may disregard one over the other or fail to engage both. Predominantly studies in this review applied a ‘one size fits all approach’ which may be detrimental to both men and women [29]. Tailored interventions can nullify these limitations, taking into account the social diversity that is apparent in young men and making applicable for all the different subgroups (e.g. fathers, unemployed, students, homosexual men, ethnic minorities) [35]. Previous research has shown gender tailoring can be effective in men [150, 151].

Early work has suggested hegemonic masculinity to be a strong predictor of risky behaviours [30] and is linked with poorer health behaviours [24–26]. However modern societal changes suggest a move away from the conventional hegemonic masculinity and a move towards a plurality of masculinities [152]. Masculinity is not a fixed entity and can change according to gender relations in a particular setting [153]. According to Mullen et al [152] young men are constructing post-modern masculine identities because of changes to youth culture, leisure patterns, employment (i.e. fewer job opportunities) and educational destinations (i.e. more young men in vocational and higher education). Therefore future research should not treat young men as one homogenous group, particularly because they are going through a range of key life events such as moving away from home [23], developing emerging sexual identities [152], starting work, parenthood and unemployment [152]. Further, this supports the need for tailored interventions specific to the unique characteristics of the individual person and by taking into account the social diversity across young men.

Theoretical frameworks are useful during the planning, implementing and evaluating interventions [154] and can maximise the chance of success, with increases in effect size for health-related behaviours including physical inactivity, poor diet, tobacco smoking and alcohol use [155]. Most of the interventions failed to list or operationalise theories. Future interventions targeting these risk behaviours should consider framing and operationalising appropriate theories to improve likelihood of success [156]. The Health, Illness, Men and Masculinities (HIMM) theoretical framework [157] considers men’s health in a larger social context and considers the influence of masculinity across the life course. In addition, the Communities of Practice Framework [158] examines how identities are learned and reproduced within certain subgroups and communities (e.g. families, workplace, sports teams, fraternities etc.) and can help to understand the shifts and discrepancies in young men’s health and risk behaviour practices. Although both frameworks are not directly constructed for young men, they do show promise and provide direction for health related interventions aimed at the social diversities apparent in this group. Although research has shown that theoretical frameworks are effective, there is some conjecture in the literature [159, 160]. The inconsistency in effectiveness may explain the lack of theoretical frameworks utilized by the included studies in this review.

Included studies predominantly recruited participants from, and conducted interventions within, a university/college setting. This is likely to be due to the large numbers of young adult males located in these settings and also where researchers are based. This therefore brings into question the generalizability of the interventions and representativeness of the samples. University samples generally come from higher socio-economic backgrounds [161], therefore it is vital to recruit and engage a diverse population sample from a wide socio-economic and cultural demography. Developing interventions that take place in settings where young men usually congregate or are located may be effective in targeting a greater number of young adult males. This is particularly evident since all of the studies that utilised a military/army setting were exclusively young adult male samples and on several occasions sample sizes of >500 were achieved [52, 60, 61, 119, 128].

The face-to-face method was the most common form of intervention delivery mode, even though this approach may be perceived as a high participant burden (e.g. associated time and travel factors) [162]. A small number of interventions used online and/or mobile phones as an intervention mode, with all of the studies using these methods published from 2008 onwards. However the best delivery mode for young men is unclear and research is required to determine reach, engagement and effect on outcomes.

Very few studies reported having male-only facilitators, even within male-only intervention studies. Early psychological research has emphasised the effects of similarity on attitude change and persuasion [163]. Facilitators who are similar to the participants in terms of sex may therefore influence intervention effectiveness. It is clear that people are more likely to disclose to others who are similar to them [164]. This may be most relevant to sexual behaviour interventions, given the sensitivity of this topic. This is supported by the greater number of male only facilitators in interventions addressing this risk behaviour in the current review and the greater number of peer-led educators. This factor may be an important element in the delivery of interventions for young men; however it was not addressed by the majority of other studies in this review. Therefore, the sex of the facilitator involved in intervention delivery should be considered in future intervention design, and reporting of research. For the interventions with no face-to-face contact in which facilitators are not required (e.g. online research), it is important to ensure the program remains sensitive to unique characteristics of the target population. Men’s only weight loss interventions developed by Morgan and colleagues [165, 166] achieved this by using preferred language of men and relevant examples in intervention materials (online and paper based). Similar approaches can be applied in interventions for young men that do not use face-to-face contact, to ensure it remains credible, relatable and likeable for the young men [167].

This scoping review has several strengths, namely: a comprehensive search strategy was implemented across multiple databases and grey literature with no date restrictions. The use of multiple risk behaviours provided a thorough synthesis of the evidence base in young adult men. Also a detailed data extraction process relative to other published scoping reviews provided comprehensive information about the included studies. Although, included studies were limited to English language papers only, it has been found that English language publications in public health are over-represented with 96.5% of 210,433 public health publications in Europe being reported in English [168]. Thus it is likely that only a small minority of studies would have been missed.

There were potentially additional studies that included young males, but results were not stratified by age and/or sex and thus we could not be certain that the population in these studies were comprised of the target group and therefore these studies were excluded. Some studies included age and sex in the regression models during analysis but were ultimately excluded for failing to specifically report intervention outcome effects in young adult males. Similarly, several studies (n = 109) were excluded as they did not provide the age range of the participants. To identify a large volume of literature, a broad age range (aged 17-35 years) was used to define young men. However, young men are not homogenous and thus different risk behaviours may be more prevalent at particular life stages [157]. For example, men who are in their 30’s may be very different to men in their late teens/early 20s’ in lifestyle factors affecting health such as; marital status, occupational status, housing environment, educational attainment and family circumstances [169]. Therefore future research should look to present results by age sub-groups to better reflect the diversity in risk behaviours among young men.

Qualitative research seeks to study social relations of human behaviour [170] and was therefore not included in this review as we were interested in the describing which risk behaviours had been addressed in young men and the details regarding the components of these interventions. However, such information can provide valuable information on perceptions of risk and usefulness/acceptability on interventions for this group. Furthermore studies were excluded if participants were from low or low-to-middle income countries because of the large heterogeneity between low-income/low-to-middle income and upper-middle/high-income countries. However, this presumes there is no difference between income and cultural groups within upper-middle/high-income countries, which is not the case and is another limitation of this review. The use of numerical analysis for the scoping review means we cannot assert whether risk taking permeates multiple practices in young men, but there is a need for future research to examine this further. Although the scoping review study design can identify large volumes of literature, they do not appraise the quality of evidence, nor do they address ‘synthesis’, that is the relative weight of evidence in favour of effectiveness of any particular interventions [46]. The database of literature identified in a scoping review can serve as a foundation for more detailed systematic reviews [171] where the quality of evidence and synthesis can be explored.

## Conclusions

Young men have been targeted to change risk behaviours to some extent, but this has not been comprehensive and is disproportionate across risk behaviours. Considering the health implications associated with young men’s lifestyle, interventions are required that exclusively target the promotion of healthy behaviours or decrease in risky behaviours in this group.

### Recommendations for researchers

-  Targeted and tailored interventions specific to young adult males are required instead of a ‘one size fits all approach’. Interventions must address young male’s personalities and behaviours during intervention development and use appropriate theoretical frameworks.
-  Future research should report mean age as well as the age range of all participants, even though this is not strictly advised in the CONSORT statement [172]. Including an age range is vital to enable a detailed indication of the sample for whom the intervention was developed.
-  If research is stratified by sex, future research must provide sample sizes for both sexes.
-  If using a diverse age range to define young men, results presented by age sub-groups will help to identify any heterogeneity in lifestyle behaviours that may be linked to risky behaviours.
-  Better reporting of effects by sex is required and possibly a series of Individual Participant Data (IPD) meta-analyses of existing trials to build on the sex database and produce more reliable results [173].
-  Future interventions targeting risk behaviours should consider recruiting representative samples given the vast majority to date are in university samples. Choosing environments where young males often congregate e.g. military/army or sporting clubs [174] and using a variety of settings may help to achieve this.
-  Risk taking is understood as a male issue, whether there are benefits in including significant other males (i.e. father-son or couple dyad interventions) need to be considered in future research.
-  The use of male only facilitators when working with young adult males should be considered and reported, given that this could be a factor affecting the intervention delivery. Researchers should also report the sex of the intervention facilitator.

## Electronic supplementary material

Additional file 1: Search strategy.(PDF 521 KB)

Additional file 2: Details of included studies.(DOCX 45 KB)

Below are the links to the authors’ original submitted files for images.

Authors’ original file for figure 1
