# Supplementary material for: A scoping review of risk behaviour interventions in young men
Source: BMC Public Health. 2014 Sep 16;14:957. doi: 10.1186/1471-2458-14-957 (PMC4177699; doi:10.1186/1471-2458-14-957)
Supplement: Supplementary file 1 — Additional file 1: Search strategy.(PDF 521 KB) [file 12889_2014_7077_MOESM1_ESM.pdf]

Additional file 1- Search strategy.

MEDLINE (Ovid)

Database(s): Ovid MEDLINE(R) 1946 to Present with Daily Update
Search Strategy:

#	Searches	Results
1	Health behavior/	30798
2	dangerous behavior/ or drinking behavior/ or drug-seeking behavior/ or risk-taking/ or sexual behavior/	61491
3	risk reduction behavior/	6377
4	exp exercise/	102579
5	Physical activity.tw.	46021
6	Nutrition.tw.	89298
7	exp diet/	183149
8	obesity/ or obesity, abdominal/ or obesity, morbid/	127047
9	Overweight/	9952
10	Ideal body weight/	90
11	exp substance-related disorders/	352970
12	alcoholism/ or binge drinking/	65116
13	Behavior, addictive/	4731
14	Addic*.tw.	36381
15	Smoking/	113533
16	Smoke-Free Policy/	26
17	drug overdose/ or substance abuse, intravenous/	18832
18	Sexually Transmitted Diseases/	19687
19	Alcohol drinking/	48687
20	"High speed driving".tw.	12
21	Violence/	23160
22	Self-Injurious Behavior/	4455
23	HIV/	15878
24	Accidents, Traffic/	33182
25	or/1-24	1087056
26	"young adult male*".tw.	1855
27	"young adult men".tw.	519
28	(young* adj3 (adult* or men or male* or man)).tw.	91301
29	young adult/ and male/	232093
30	college aged men.tw. or students/	31967
31	or/26-30	339582
32	clinical trial/ or clinical trial, phase i/ or clinical trial, phase ii/ or clinical trial,	722776

	phase iii/ or clinical trial, phase iv/ or controlled clinical trial/ or multicenter study/ or randomized controlled trial/	
33	exp Clinical Trials as Topic/	267472
34	cross-over studies/ or double-blind method/ or single-blind method/ or matched-pair analysis/ or random allocation/ or control groups/	228089
35	(pre post or pre test or post test or non randomi?ed or quasi experiment).tw.	14223
36	Feasibility studies/	39607
37	Intervention Studies/	5966
38	pilot projects/	77027
39	(random* adj3 (study or studies or trial or trials)).tw.	223258
40	(random* adj3 (allocation or assign* or allocate*)).tw.	82531
41	((blind* or mask*) adj2 (singl* or doubl* or trebl* or tripl*)).tw.	118598
42	(control adj group*).tw.	248383
43	((trial or trials) adj2 (clinical or controlled)).tw.	276822
44	("outcome study" or "outcome studies" or quasiexperimental or "quasi experimental" or quasi-experimental or "pseudo experimental").tw.	9745
45	((cluster or factorial) adj2 trial*).tw.	1758
46	Meta-analysis/ or cross-over studies/	72334
47	(meta-analysis or crossover* or "cross over*" or cross-over*).tw.	92109
48	intervention.tw.	282022
49	or/32-48	1622456
50	25 and 31 and 49	13605
51	addresses/ or lectures/ or anecdotes/ or biography/ or interview/ or comment/ or directory/ or editorial/ or legal cases/ or case reports/ or legislation/ or letter/ or news/ or newspaper article/ or patient education handout/	2986309
52	50 not 51	13479
53	limit 52 to (english language and male and humans and ("adolescent (13 to 18 years)" or "young adult (19 to 24 years)"))	10492
54	"Aged, 80 and over"/ or Aged/ or Middle Aged/	3665932
55	53 not 54	5920

EMBASE (Ovid)

Database(s): Embase Classic+Embase 1947 to 2013 May 14
Search Strategy:

#	Searches	Results
1	Health behavior/	38645
2	drinking behavior/ or drug-seeking behavior/ or high risk behavior/ or sexual behavior/	124776
3	risk reduction/	53217
4	exp exercise/	211660
5	Physical activity.tw.	64195
6	overnutrition/	3029
7	exp diet/	223241
8	obesity/ or abdominal obesity/ or morbid obesity/	243436
9	body weight/	195624
10	alcoholism/	104247
11	addiction/	45343
12	addic*.tw.	55816
13	smoking/	156424
14	drug overdose/	16228
15	substance abuse/	36331
16	sexually transmitted disease/	36761
17	alcohol consumption/	65906
18	"high speed driving".tw.	24
19	violence/	33842
20	automutilation/	10460
21	Human immunodeficiency virus/	61617
22	traffic accident/	45857
23	or/1-22	1485937
24	"young adult male*".tw.	2597
25	"young adult men".tw.	630
26	(young* adj3 (adult* or men or male* or man)).tw.	126087
27	college aged men.tw. or college student/ or student/	60647
28	or/24-27	184904
29	controlled clinical trial/ or clinical trial/ or randomized controlled trial/ or multicenter study/ or "phase 2 clinical trial (topic)"/ or "phase 4 clinical trial (topic)"/ or "phase 3 clinical trial (topic)"/ or "phase 1 clinical trial (topic)"/	967133

30	exp "clinical trial (topic)"/	61439
31	crossover procedure/ or double blind procedure/ or single blind procedure/ or control group/ or randomization/	269289
32	(pre post or pre test or post test or non randomi?ed or quasi experiment).tw.	22725
33	feasibility study/	45414
34	intervention study/	15960
35	pilot study/	65093
36	(random* adj3 (study or studies or trial or trials)).tw.	307863
37	(random* adj3 (allocation or assign* or allocate*)).tw.	105279
38	((blind* or mask*) adj2 (singl* or doubl* or trebl* or tripl*)).tw.	162361
39	(control adj group*).tw.	362741
40	((trial or trials) adj2 (clinical or controlled)).tw.	389537
41	("outcome study" or "outcome studies" or quasiexperimental or "quasi experimental" or quasi-experimental or "pseudo experimental").tw.	13308
42	((cluster or factorial) adj2 trial*).tw.	2248
43	Meta-analysis/ or crossover procedure/	107787
44	(meta-analysis or crossover* or "cross over*" or cross-over*).tw.	123214
45	intervention.tw.	403562
46	or/29-45	2089648
47	23 and 28 and 46	6562
48	addresses/ or lectures/ or anecdotes/ or biography/ or interview/ or comment/ or directory/ or editorial/ or legal cases/ or case reports/ or legislation/ or letter/ or news/ or newspaper article/ or patient education handout/	1496198
49	47 not 48	6286
50	limit 49 to (english language and male and humans)	3729
51	"Aged, 80 and over"/ or Aged/ or Middle Aged/	2729213
52	50 not 51	3336

MEDLINE in process (Ovid)

Search History (55 searches) (Click to close)

	# ▲	Searches	Results
	1	Health behavior/	0
	2	dangerous behavior/ or drinking behavior/ or drug-seeking behavior/ or risk-taking/ or sexual behavior/	0
	3	risk reduction behavior/	0
	4	exp exercise/	0
	5	Physical activity.tw.	4011
	6	Nutrition.tw.	6017
	7	exp diet/	0
	8	obesity/ or obesity, abdominal/ or obesity, morbid/	0
	9	Overweight/	0
	10	Ideal body weight/	0
	11	exp substance-related disorders/	1
	12	alcoholism/ or binge drinking/	0
	13	Behavior, addictive/	0
	14	Addic*.tw.	2371
	15	Smoking/	1
	16	Smoke-Free Policy/	0
	17	drug overdose/ or substance abuse, intravenous/	0
	18	Sexually Transmitted Diseases/	0
	19	Alcohol drinking/	0
	20	"High speed driving".tw.	0
	21	Violence/	0

☐	22	Self-Injurious Behavior/	0
☐	23	HIV/	0
☐	24	Accidents, Traffic/	0
☐	25	or/1-24	12029
☐	26	"young adult male*".tw.	85
☐	27	"young adult men".tw.	20
☐	28	(young* adj3 (adult* or men or male* or man)).tw.	5428
☐	29	young adult/ and male/	2
☐	30	college aged men.tw. or students/	10
☐	31	or/26-30	5440
☐	32	clinical trial/ or clinical trial, phase i/ or clinical trial, phase ii/ or clinical trial, phase iii/ or clinical trial, phase iv/ or controlled clinical trial/ or multicenter study/ or randomized controlled trial/	950
☐	33	exp Clinical Trials as Topic/	4
☐	34	cross-over studies/ or double-blind method/ or single-blind method/ or matched-pair analysis/ or random allocation/ or control groups/	1
☐	35	(pre post or pre test or post test or non randomi?ed or quasi experiment).tw.	1405
☐	36	Feasibility studies/	0
☐	37	Intervention Studies/	0
☐	38	pilot projects/	0
☐	39	(random* adj3 (study or studies or trial or trials)).tw.	16176
☐	40	(random* adj3 (allocation or assign* or allocate*)).tw.	5151
☐	41	((blind* or mask*) adj2 (singl* or doubl* or trebl* or tripl*)).tw.	4875
☐	42	(control adj group*).tw.	17191
☐	43	((trial or trials) adj2 (clinical or controlled)).tw.	21066
☐	44	("outcome study" or "outcome studies" or quasiexperimental or "quasi experimental" or quasi-experimental or "pseudo experimental").tw.	784
☐	45	((cluster or factorial) adj2 trial*).tw.	220
☐	46	Meta-analysis/ or cross-over studies/	76
☐	47	(meta-analysis or crossover* or "cross over*" or cross-over*).tw.	9488
☐	48	intervention.tw.	22009

☐	49	or/32-48	71034
☐	50	25 and 31 and 49	39
☐	51	addresses/ or lectures/ or anecdotes/ or biography/ or interview/ or comment/ or directory/ or editorial/ or legal cases/ or case reports/ or legislation/ or letter/ or news/ or newspaper article/ or patient education handout/	72481
☐	52	50 not 51	38
☐	53	limit 52 to (english language and male and humans and ("adolescent (13 to 18 years)" or "young adult (19 to 24 years)"))	0
☐	54	"Aged, 80 and over"/ or Aged/ or Middle Aged/	6
☐	55	53 not 54	0

PsycINFO (Ovid)

Database(s): PsycINFO 1806 to May Week 1 2013
Search Strategy:

#	Searches	Results
1	health behavior/	14585
2	drinking behavior/ or risk taking/ or sexual risk taking/	14810
3	risk reduction behavio?r.tw.	63
4	exp exercise/	15218
5	physical activity.tw.	15711
6	Nutrition/	6166
7	diets/	8289
8	obesity/ or overweight/	14510
9	drug abuse/	33697
10	alcoholism/ or alcohol abuse/	35573
11	addiction/	5566
12	addic*.tw.	33715
13	tobacco smoking/	20890
14	smoking cessation/	8185
15	drug overdoses/ or drug abuse/	34563
16	sexually transmitted diseases/	3225
17	exp Alcohol Drinking Patterns/	50520
18	driving behavior/ or driving under the influence/	7878
19	"high speed driving".tw.	9
20	Violence/	21499
21	Self Injurious Behavior/	1390
22	HIV/	23629
23	Motor Traffic Accidents/	4093
24	or/1-23	240944
25	"young adult male*".tw.	717
26	"young adult men".tw.	183
27	(young* adj3 (adult* or men or male* or man)).tw.	44301
28	college students/ or students/	71155
29	college aged men.tw.	42
30	or/25-29	114289

31	clinical trial/ or clinical study/ or controlled clinical trial/ or multicenter study/ or phase 1 clinical trial/ or phase 2 clinical trial/ or phase 3 clinical trial/ or phase 4 clinical trial/ or randomized controlled trial/	6719
32	exp "clinical trial (topic)"/	0
33	randomization/ or control group/	656
34	feasibility study/	0
35	intervention study/	0
36	pilot study/	0
37	(pre post or pre test or post test or non randomi?ed or quasi experiment).tw.	9048
38	(random* adj3 (study or studies or trial or trials)).tw.	29568
39	(random* adj3 (allocation or assign* or allocate*)).tw.	27572
40	((blind* or mask*) adj2 (singl* or doubl* or trebl* or tripl*)).tw.	18518
41	(control adj group*).tw.	52992
42	((trial or trials) adj2 (clinical or controlled)).tw.	36307
43	("outcome study" or "outcome studies" or quasiexperimental or "quasi experimental" or quasi-experimental or "pseudo experimental").tw.	9332
44	((cluster or factorial) adj2 trial*).tw.	336
45	meta analysis/	3283
46	(meta-analysis or crossover* or "cross over*" or cross-over*).tw.	19417
47	intervention.tw.	139161
48	or/31-47	262895
49	24 and 30 and 48	1986
50	addresses/ or lectures/ or anecdotes/ or biography/ or interview/ or comment/ or directory/ or editorial/ or legal cases/ or case reports/ or legislation/ or letter/ or news/ or newspaper article/ or patient education handout/	5373
51	49 not 50	1986
52	limit 51 to (human and male and english language)	1210
53	"Aged, 80 and over"/ or Aged/ or Middle Aged/	1568
54	52 not 53	1210

Science Citation Index (WoS)

Search History

Set	Results	Save History / Create Alert Open Saved History
# 38	4,524	(#36 AND #25 AND #20) AND Language=(English) AND Document Types=(Article OR Book Chapter OR Meeting Abstract OR Proceedings Paper OR Reprint OR Review)<br>Databases=SCI-EXPANDED, CCR-EXPANDED, IC Timespan=All years
# 37	4,716	#36 AND #25 AND #20<br>Databases=SCI-EXPANDED, CCR-EXPANDED, IC Timespan=All years
# 36	971,069	#35 OR #34 OR #33 OR #32 OR #31 OR #30 OR #29 OR #28 OR #27 OR #26<br>Databases=SCI-EXPANDED, CCR-EXPANDED, IC Timespan=All years
# 35	51,539	TS=(meta-analysis)<br>Databases=SCI-EXPANDED, CCR-EXPANDED, IC Timespan=All years
# 34	555,752	TS=("control group*") OR TS=("clinical trial" or "clinical trials" or "controlled trial" or "controlled trials")<br>Databases=SCI-EXPANDED, CCR-EXPANDED, IC Timespan=All years
# 33	140,195	TS=("outcome study" or "outcome studies" or quasiexperimental or "quasi experimental" or quasi-experimental or "pseudo experimental") OR TS=(meta-analysis or crossover* or "cross over*" or cross-over*) OR TS=("cluster design*" or "factorial design*" "cluster trial*" or "factorial trial*")<br>Databases=SCI-EXPANDED, CCR-EXPANDED, IC Timespan=All years
# 32	183,554	TS=(((blind* or mask*) SAME (singl* or doubl* or trebl* or tripl*)))<br>Databases=SCI-EXPANDED, CCR-EXPANDED, IC Timespan=All years
# 31	93,767	TS=(random* SAME (allocation or assign* or allocate*))<br>Databases=SCI-EXPANDED, CCR-EXPANDED, IC Timespan=All years
# 30	135,113	TS=("random* study" or "random* studies" or "random* trial" or "random* trials")<br>Databases=SCI-EXPANDED, CCR-EXPANDED, IC Timespan=All years
# 29	72,585	TS= ("Feasibility stud*" or "Intervention Stud*" or "pilot project*" or "pilot stud*")<br>Databases=SCI-EXPANDED, CCR-EXPANDED, IC Timespan=All years
# 28	12,631	TS=("pre post" or "pre test" or "post test" or "non randomi?ed" or "quasi experiment")<br>Databases=SCI-EXPANDED, CCR-EXPANDED, IC Timespan=All years
# 27	36,071	TS= ("cross-over studies" or "double-blind method" or "single-blind method" or "matched-pair analysis" or "random allocation" or "control groups")<br>Databases=SCI-EXPANDED, CCR-EXPANDED, IC Timespan=All years
# 26	112,422	TS=("multicenter study" or "multicentre study" or "clinical trial" or "controlled clinical trial" "randomized controlled trial")<br>Databases=SCI-EXPANDED, CCR-EXPANDED, IC Timespan=All years

# 25	197,029	#24 OR #23 OR #22 OR #21<br>Databases=SCI-EXPANDED, CCR-EXPANDED, IC Timespan=All years
# 24	131,077	TS= ("college aged men" or student*)<br>Databases=SCI-EXPANDED, CCR-EXPANDED, IC Timespan=All years
# 23	68,748	TS= ("Young adult*" or "young men" or "young male*" or "young man")<br>Databases=SCI-EXPANDED, CCR-EXPANDED, IC Timespan=All years
# 22	435	TS=("young adult men")<br>Databases=SCI-EXPANDED, CCR-EXPANDED, IC Timespan=All years
# 21	1,334	TS=("young adult male*")<br>Databases=SCI-EXPANDED, CCR-EXPANDED, IC Timespan=All years
# 20	1,198,028	#19 OR #18 OR #17 OR #16 OR #15 OR #14 OR #13 OR #12 OR #11 OR #10 OR #9 OR #8 OR #7 OR #6 OR #5 OR #4 OR #3 OR #2 OR #1<br>Databases=SCI-EXPANDED, CCR-EXPANDED, IC Timespan=All years
# 19	3,106	TS= ("accidents, traffic" or "traffic accidents")<br>Databases=SCI-EXPANDED, CCR-EXPANDED, IC Timespan=All years
# 18	235,869	TS= (HIV or "Human immunodeficiency virus")<br>Databases=SCI-EXPANDED, CCR-EXPANDED, IC Timespan=All years
# 17	992	TS=("Self-Injurious Behavior" OR "Self-Injurious Behaviour")<br>Databases=SCI-EXPANDED, CCR-EXPANDED, IC Timespan=All years
# 16	16,806	TS=(Violence)<br>Databases=SCI-EXPANDED, CCR-EXPANDED, IC Timespan=All years
# 15	41	TS=("high speed driving")<br>Databases=SCI-EXPANDED, CCR-EXPANDED, IC Timespan=All years
# 14	23,314	TS=(Alcohol drinking)<br>Databases=SCI-EXPANDED, CCR-EXPANDED, IC Timespan=All years
# 13	11,105	TS=("Sexually transmitted disease*")<br>Databases=SCI-EXPANDED, CCR-EXPANDED, IC Timespan=All years
# 12	927	TS= ("drug overdose" or "substance abuse, intravenous")<br>Databases=SCI-EXPANDED, CCR-EXPANDED, IC Timespan=All years
# 11	157,703	TS=(Smoking or "smoke-free policy")<br>Databases=SCI-EXPANDED, CCR-EXPANDED, IC Timespan=All years
# 10	33,788	TS=(addic*)<br>Databases=SCI-EXPANDED, CCR-EXPANDED, IC Timespan=All years
# 9	625	TS=("Behavior, addictive" or "addictive behavior" or "Behaviour, addictive" or "addictive behaviour")<br>Databases=SCI-EXPANDED, CCR-EXPANDED, IC Timespan=All years
# 8	21,124	TS= (alcoholism or "binge drinking")<br>Databases=SCI-EXPANDED, CCR-EXPANDED, IC Timespan=All years
# 7	541	TS= ("substance-related disorders")<br>Databases=SCI-EXPANDED, CCR-EXPANDED, IC Timespan=All years
# 6	160,489	TS= (Obesity or "obesity, abdominal", or "obesity, morbid" or overweight, or "ideal body weight")<br>Databases=SCI-EXPANDED, CCR-EXPANDED, IC Timespan=All years
# 5	385,594	TS=(Diet or nutrition)<br>Databases=SCI-EXPANDED, CCR-EXPANDED, IC Timespan=All years

# 4	258,526	TS=(Exercise or "Physical activity")<br>Databases=SCI-EXPANDED, CCR-EXPANDED, IC Timespan=All years
# 3	4,728	TS=("risk behavior" or "risk behaviour" or "risk behavior change" or "risk behaviour change" or "risk reduction behavior" "risk reduction behaviour")<br>Databases=SCI-EXPANDED, CCR-EXPANDED, IC Timespan=All years
# 2	21,337	TS= ("dangerous behavior" or "drinking behavior" or "drug-seeking behavior" or "risk-taking" or "sexual behavior" or "dangerous behaviour" or "drinking behaviour" or "drug-seeking behaviour" or "risk-taking" or "sexual behaviour")<br>Databases=SCI-EXPANDED, CCR-EXPANDED, IC Timespan=All years
# 1	4,291	TS= ("Health behavior" or "health behaviour")<br>Databases=SCI-EXPANDED, CCR-EXPANDED, IC Timespan=All years

Cinahl (EbscoHost)

Revised Date: 07/2011
Wednesday, May 15, 2013 12:06:15 AM

#	Query	Limiters/Expanders	Results
S53	S26 AND S33 AND S52	Search modes - Boolean/Phrase	3,355
S52	S34 OR S35 OR S36 OR S37 OR S38 OR S39 OR S40 OR S41 OR S42 OR S43 OR S44 OR S45 OR S46 OR S47 OR S48 OR S49 OR S50 OR S51	Search modes - Boolean/Phrase	367,979
S51	TI intervention OR AB intervention	Search modes - Boolean/Phrase	92,626
S50	TI ((meta-analysis or crossover* or "cross over*" or cross-over*)) OR AB ((meta-analysis or crossover* or "cross over*" or cross-over*))	Search modes - Boolean/Phrase	20,772
S49	TI ((meta-analysis or crossover* or "cross over*" or cross-over*)) OR AB ((meta-analysis or crossover* or "cross over*" or cross-over*))	Search modes - Boolean/Phrase	20,772
S48	(MH "Meta Analysis")	Search modes - Boolean/Phrase	16,345
S47	TI (((cluster or factorial) N2 trial*)) OR AB (((cluster or factorial) N2 trial*))	Search modes - Boolean/Phrase	1,743
S46	TI (("outcome study" or "outcome studies" or quasiexperimental or "quasi experimental" or quasi-experimental or "pseudo experimental")) OR AB (("outcome study" or "outcome studies" or quasiexperimental or "quasi experimental" or quasi-experimental or "pseudo experimental"))	Search modes - Boolean/Phrase	5,228
S45	TI (((trial or trials) N2 (clinical or controlled))) OR AB (((trial or trials) N2 (clinical or controlled)))	Search modes - Boolean/Phrase	71,421
S44	TI (control N1 group*) OR AB (control N1 group*)	Search modes - Boolean/Phrase	32,878
S43	TI (((blind* or mask*) N2 (singl* or doubl* or trebl* or tripl*))) OR AB (((blind* or mask*) N2 (singl* or doubl* or trebl* or tripl*)))	Search modes - Boolean/Phrase	18,501
S42	TI ((random* N3 (allocation or assign* or allocate*))) OR AB ((random* N3 (allocation or assign* or allocate*)))	Search modes - Boolean/Phrase	17,981

S41	TI ((random* N3 (study or studies or trial or trials))) OR AB ((random* N3 (study or studies or trial or trials)))	Search modes - Boolean/Phrase	70,586
S40	(MH "Intervention Trials")	Search modes - Boolean/Phrase	4,847
S39	(MH "Pilot Studies")	Search modes - Boolean/Phrase	38,455
S38	TI "feasibility studies" OR AB "feasibility studies"	Search modes - Boolean/Phrase	77
S37	(MH "Experimental Studies")	Search modes - Boolean/Phrase	14,247
S36	TI ((pre post or pre test or post test or non randomi?ed or quasi experiment)) OR AB ((pre post or pre test or post test or non randomi?ed or quasi experiment))	Search modes - Boolean/Phrase	13,846
S35	(MH "Crossover Design") OR (MH "Double-Blind Studies") OR (MH "Single-Blind Studies") OR (MH "Triple-Blind Studies") OR (MH "Matched-Pair Analysis") OR (MH "Random Assignment") OR (MH "Control Group")	Search modes - Boolean/Phrase	70,269
S34	(MH "Clinical Trials") OR (MH "Pretest-Posttest Design") OR (MH "Randomized Controlled Trials") OR (MH "Preventive Trials") OR (MH "Nonrandomized Trials") OR (MH "Multicenter Studies")	Search modes - Boolean/Phrase	158,382
S33	S27 OR S28 OR S29 OR S30 OR S31 OR S32	Search modes - Boolean/Phrase	74,926
S32	TI "college aged men" OR AB "college aged men"	Search modes - Boolean/Phrase	58
S31	(MH "Students")	Search modes - Boolean/Phrase	7,777
S30	(MH "Young Adult") AND (MH "Male")	Search modes - Boolean/Phrase	51,486
S29	TI ((young* N3 (adult* or men or male* or man))) OR AB ((young* N3 (adult* or men or male* or man)))	Search modes - Boolean/Phrase	19,087
S28	TI "young adult men" OR AB "young adult men"	Search modes - Boolean/Phrase	95
S27	TI "young adult male*" OR AB "young adult male*"	Search modes - Boolean/Phrase	191
S26	S1 OR S2 OR S3 OR S4 OR S5 OR S6 OR S7 OR S8 OR S9 OR S10 OR S11 OR S12 OR S13 OR S14 OR S15 OR S16 OR S17 OR S18 OR S19 OR S20 OR S21 OR S22 OR S23 OR S24 OR S25	Search modes - Boolean/Phrase	318,632

S25	(MH "Accidents, Traffic")	Search modes - Boolean/Phrase	9,832
S24	(MH "Human Immunodeficiency Virus")	Search modes - Boolean/Phrase	2,806
S23	(MH "Self-Injurious Behavior")	Search modes - Boolean/Phrase	1,923
S22	(MH "Violence")	Search modes - Boolean/Phrase	10,593
S21	TI "high speed driving" OR AB "high speed driving"	Search modes - Boolean/Phrase	1
S20	(MH "Alcohol Drinking")	Search modes - Boolean/Phrase	14,876
S19	(MH "Sexually Transmitted Diseases")	Search modes - Boolean/Phrase	8,113
S18	(MH "Overdose") OR (MH "Substance Abuse, Intravenous")	Search modes - Boolean/Phrase	5,610
S17	(MH "Smoking")	Search modes - Boolean/Phrase	32,868
S16	TI addic* OR AB addic*	Search modes - Boolean/Phrase	9,435
S15	(MH "Behavior, Addictive")	Search modes - Boolean/Phrase	2,677
S14	(MH "Alcoholism") OR (MH "Alcohol Abuse")	Search modes - Boolean/Phrase	15,993
S13	(MH "Substance Use Disorders+")	Search modes - Boolean/Phrase	88,965
S12	(MH "Body Weight Changes")	Search modes - Boolean/Phrase	96
S11	TI overweight OR AB overweight OR TI Abdominal obesity OR AB abdominal obesity	Search modes - Boolean/Phrase	10,745
S10	(MH "Obesity") OR (MH "Obesity, Morbid")	Search modes - Boolean/Phrase	46,017
S9	(MH "Diet+")	Search modes - Boolean/Phrase	58,271
S8	(MH "Nutrition")	Search modes - Boolean/Phrase	15,831
S7	TI physical activity OR AB physical activity	Search modes - Boolean/Phrase	20,465
S6	(MH "Exercise+")	Search modes - Boolean/Phrase	58,527
S5	TI risk reduction OR AB risk reduction	Search modes -	5,543

		Boolean/Phrase	
S4	(MH "Risk Management (Iowa NIC) (Non-Cinahl)")	Search modes - Boolean/Phrase	0
S3	TI dangerous behavio?r OR AB dangerous behavio?r OR TI drug- seeking behavio?r OR AB drug- seeking behavio?r	Search modes - Boolean/Phrase	38
S2	(MH "Drinking Behavior") OR (MH "Risk Taking Behavior") OR (MH "Sexual Behavior Analysis (Saba CCC)")	Search modes - Boolean/Phrase	12,079
S1	MH health behavior	Search modes - Boolean/Phrase	24,823

Cochrane Library (Wiley)

ID	Search	Hits
#1	MeSH descriptor: [Health Behavior] this term only	1958
#2	MeSH descriptor: [Dangerous Behavior] this term only	30
#3	MeSH descriptor: [Drinking Behavior] this term only	70
#4	MeSH descriptor: [Drug-Seeking Behavior] this term only	6
#5	MeSH descriptor: [Risk-Taking] this term only	792
#6	MeSH descriptor: [Sexual Behavior] this term only	1204
#7	MeSH descriptor: [Risk Reduction Behavior] this term only	805
#8	MeSH descriptor: [Exercise] explode all trees	11839
#9	physical activity:ti,ab,kw (Word variations have been searched)	8790
#10	"nutrition":ti,ab,kw (Word variations have been searched)	8261
#11	MeSH descriptor: [Diet] explode all trees	10672
#12	MeSH descriptor: [Obesity] 1 tree(s) exploded	6407
#13	MeSH descriptor: [Overweight] this term only	1052
#14	MeSH descriptor: [Ideal Body Weight] this term only	4
#15	MeSH descriptor: [Substance-Related Disorders] explode all trees	10604
#16	MeSH descriptor: [Alcoholism] this term only	2199
#17	MeSH descriptor: [Binge Drinking] this term only	1
#18	MeSH descriptor: [Behavior, Addictive] this term only	277
#19	addic*:ti,ab,kw (Word variations have been searched)	2066
#20	MeSH descriptor: [Smoking] this term only	4785
#21	MeSH descriptor: [Smoke-Free Policy] this term only	1
#22	MeSH descriptor: [Drug Overdose] this term only	5
#23	MeSH descriptor: [Substance Abuse, Intravenous] this term only	327
#24	MeSH descriptor: [Sexually Transmitted Diseases] this term only	561
#25	MeSH descriptor: [Alcohol Drinking] this term only	2126
#26	"high speed driving":ti,ab,kw (Word variations have been searched)	1
#27	MeSH descriptor: [Violence] this term only	344
#28	MeSH descriptor: [Self-Injurious Behavior] this term only	174
#29	MeSH descriptor: [HIV] this term only	304
#30	MeSH descriptor: [Accidents, Traffic] this term only	343
#31	(#1 or #2 or #3 or #4 or #5 or #6 or #7 or #8 or #9 or #10 or #11 or #12 or #13 or #14 or #15 or #16 or #17 or #18 or #19 or #20 or #21 or #22 or #23 or #24 or #25 or #26 or #27 or #28 or #29 or #30)	57079
#32	"Young adult male*":ti,ab,kw (Word variations have been searched)	112
#33	"young adult men" .tw.:ti,ab,kw (Word variations have been searched)	0
#34	(young* near/3 (adult* or men or male* or man)):ti,ab,kw (Word variations have been searched)	23557
#35	MeSH descriptor: [Young Adult] this term only	105
#36	MeSH descriptor: [Adolescent] this term only	69709
#37	MeSH descriptor: [Male] explode all trees	415
#38	(#35 and #37 or #36 and #37)	126

#39	MeSH descriptor: [Students] this term only	1079
#40	"college aged men":ti,ab,kw (Word variations have been searched)	41
#41	(#32 or #33 or #34 or #35 or #38 or #39 or #40)	24497
#42	(clinical trial):pt	295895
#43	(clinical trial phase i):pt or (clinical trial phase ii):pt or (clinical trial phase iii):pt or (clinical trial phase iv):pt	12625
#44	(Randomized Controlled Trial):pt	319842
#45	(Controlled Clinical Trial):pt	295195
#46	(multicenter study):pt	51044
#47	MeSH descriptor: [Clinical Trials as Topic] explode all trees	53584
#48	MeSH descriptor: [Double-Blind Method] this term only	98777
#49	MeSH descriptor: [Single-Blind Method] this term only	10903
#50	MeSH descriptor: [Control Groups] this term only	96
#51	MeSH descriptor: [Random Allocation] this term only	20407
#52	MeSH descriptor: [Matched-Pair Analysis] this term only	537
#53	MeSH descriptor: [Feasibility Studies] this term only	2756
#54	MeSH descriptor: [Intervention Studies] this term only	1743
#55	MeSH descriptor: [Pilot Projects] this term only	10010
#56	MeSH descriptor: [Cross-Over Studies] this term only	23535
#57	(pre post or pre test or post test or non randomi?ed or quasi experiment):ti,ab,kw	48840
#58	(random* near/3 (study or studies or trial or trials)):ti,ab,kw	193982
#59	(random* near/3 (allocation or assign* or allocate*)):ti,ab,kw	87193
#60	((blind* or mask*) near/2 (singl* or doubl* or trebl* or tripl*)):ti,ab,kw	162715
#61	(control next group*):ti,ab,kw	52824
#62	((trial or trials) near/2 (clinical or controlled)):ti,ab,kw	177184
#63	("outcome study" or "outcome studies" or quasiexperimental or "quasi experimental" or quasi-experimental or "pseudo experimental"):ti,ab,kw	1858
#64	((cluster or factorial) near/2 trial*):ti,ab,kw	1397
#65	MeSH descriptor: [Meta-Analysis] this term only	92
#66	(meta-analysis or crossover* or "cross over*" or cross-over*):ti,ab,kw	63774
#67	(#42 or #43 or #44 or #45 or #46 or #47 or #48 or #49 or #50 or #51 or #52 or #53 or #54 or #55 or #56 or #57 or #58 or #59 or #60 or #61 or #62 or #63 or #64 or #65 or #66)	534785
#68	(#31 and #41 and #67)	4908
#69	(addresses):pt or (lectures):pt or (anecdotes):pt or (biography):pt or (interview):pt or (comment):pt or (directory):pt or (editorial):pt or (legal cases):pt or (case reports):pt or (legislation):pt or (letter):pt or (news):pt or (newspaper article):pt or (patient education handout):pt	7978
#70	(#68 not #69)	4894
#71	MeSH descriptor: [Middle Aged] this term only	235
#72	MeSH descriptor: [Aged] this term only	192
#73	MeSH descriptor: [Aged, 80 and over] this term only	51
#74	(#71 or #72 or #73)	295
#75	(#70 not #74)	4890
