# Supplementary material for: A scoping review of risk behaviour interventions in young men
Source: BMC Public Health. 2014 Sep 16;14:957. doi: 10.1186/1471-2458-14-957 (PMC4177699; doi:10.1186/1471-2458-14-957)
Supplement: Supplementary file 2 — Additional file 2: Details of included studies.(DOCX 45 KB) [file 12889_2014_7077_MOESM2_ESM.docx]

| **Author(s) and title** | **Year** | **Study Design** | **Country** | **Target population group** | **Age range (years)** | **Risk behaviour** |
| --- | --- | --- | --- | --- | --- | --- |
| Ahola, R.. Pyky, R. Jamsa, T. Mantysaari, M. Koskimaki, H. Ikaheimo, T.M. Huotari, M.L. Roning, J. Heikkinen, H.I. Korpelainen, R.. *"Gamified physical activation of young men--a Multidisciplinary Population-Based Randomized Controlled Trial (MOPO study)* | 2013 | RCT | Finland | Young adult males only | 18 only | Physical inactivity |
| Akbarpour, M.. "*The effect of aerobic training on serum adiponectin and leptin levels and inflammatory markers of coronary heart disease in obese men."* | 2013 | RCT | Finland | Young adult males only | 20-25 | Physical inactivity |
| Anshel, M. H. *"Effect of chronic aerobic exercise and progressive relaxation on motor performance and affect following acute stress."* | 1996 | RCT | Australia | Young adult males only | 19-25 | Physical inactivity |
| Antunes, M. C. Stall, R. D. Paiva, V. Peres, C. A. Paul, J. Hudes, M. Hearst, N. “*Evaluating an AIDS sexual risk reduction program for young adults in public night schools in Sao Paulo, Brazil.”* | 1997 | Cluster RCT | Brazil | Young adults (Stratified by young males) | 18-25 | Unsafe sexual behaviour |
| Armstrong, B.J Kalmuss, D. Franks, M. Hecker, G. Bell, D. *"Creating teachable moments: a clinic-based intervention to improve young men's sexual health."* | 2010 | Case series | USA | Young adult males only | 18-30 | Unsafe sexual behaviour |
| Asci, F.H *"Sex differences in psychological effects of exercise."* | 2002 | RCT | Turkey | Young adults (Stratified by young males) | 18-27 | Physical inactivity |
| Asci, F. H. *"The effects of step dance on physical self-perception of female and male university students."* | 2009 | RCT | Turkey | Young adults (Stratified by young males) | 18-27 | Physical inactivity |
| Baer, J. S. Kivlahan, D. R. Blume, A. W. McKnight, P. Marlatt, G. A. *“Brief intervention for heavy-drinking college students: 4-year follow-up and natural history.”* | 2001 | RCT | USA | Young adults (Stratified by young males) | 18-19 | Alcohol use |
| Barnett, N.P. Apodaca, T.R. Magill, M Colby, S.M. Gwaltney, C Rohsenow, D.J. Monti, PM. *"Moderators and mediators of two brief interventions for alcohol in the emergency department."* | 2009 | RCT | USA | Young adults (Stratified by young males) | 18-24 | Alcohol use |
| Berecz, J. M. *“Reduction of cigarette smoking through self-administered aversion conditioning: a new treatment model with implications for public health”* | 1972 | RCT | USA | Young adults (Stratified by young males) | 17-30 | Tobacco smoking |
| Bingham, C. Barretto, A.I Walton, M.A. Bryant, C.M. Shope, JT. Raghunathan, T.E. *"Efficacy of a Web-based, tailored, alcohol prevention/intervention program for college students: Initial findings."* | 2010 | Cluster RCT | USA | Young adults (Stratified by young males) | 18-20 | Alcohol use |
| Bingham, C. Barretto, A.I Walton, M.A. Bryant, C.M. Shope, JT. Raghunathan, T.E. *"Efficacy of a Web-Based, Tailored, Alcohol Prevention/Intervention Program for College Students: 3-Month Follow-Up."* | 2011 | Cluster controlled | USA | Young adults (Stratified by young males) | 18-20 | Alcohol use |
| Bingham, T. & Carlos-Henderson, J. “*Evaluating Locally Developed Homegrown HIV Prevention Interventions”* | Not published yet (Grey Literature) | RCT | USA | Young adult males only | 18-29 | Unsafe sexual behaviour |
| Blow, F.C. Barry, K.L. Walton, M.A. Maio, R.F. Chermack, S.T. Bingham, C. Ignacio, R.V. Strecher, VJ. "*The Efficacy of Two Brief Intervention Strategies Among Injured, At-Risk Drinkers in the Emergency Department: Impact of Tailored Messaging and Brief Advice."* | 2006 | RCT | USA | Adults (Stratified by young males) | 19-22 | Alcohol use |
| Bond, V. Bartels, M. N. Sloan, R. P. Millis, R. M. Zion, A. S. Andrews, N. De Meersman, R. E. *"Exercise training favourably affects autonomic and blood pressure responses during mental and physical stressors in African-American men."* | 2009 | RCT | USA | Young adult males only | 18-26 | Physical inactivity |
| Bond, V. Stephens, Q. Adams, R. G. Vaccaro, P. Demeersman, R. Williams, D. Obisesan, T. O. Franks, B. D. Oke, L. M. Coleman, B. Blakely, R. Millis, R. M. *"Aerobic exercise attenuates an exaggerated exercise blood pressure response in normotensive young adult African-American men."* | 2002 | Case series | USA | Young adult males only | 18-26 | Physical inactivity |
| Borgia, P. Marinacci, C. Schifano, P. Perucci, C. A. *"Is peer education the best approach for HIV prevention in schools? Findings from a randomized controlled."* | 2005 | Cluster RCT | Italy | Young adults (Stratified by young males) | 18-19 | Unsafe sexual behaviour |
| Calfas, K. J. Sallis, J. F. Nichols, J. F. Sarkin, J. A. Johnson, M. F. Caparosa, S. Thompson, S. Gehrman, C. A. Alcaraz, J. E. “*Project GRAD: Two-year outcomes of a randomized controlled physical activity intervention among young adults.”* | 2000 | RCT | USA | Young adults (Stratified by young males) | 18-29 | Physical inactivity |
| Cambien, F. Richard, J. L. Ducimetiere, P. Warnet, J. M. Kahn, J. *“The Paris Cardiovascular Risk Factor Prevention Trial. Effects of two years of intervention in a population of young men.”* | 1981 | RCT | France | Young adult males only | 25-35 | Poor diet, Physical inactivity, Tobacco smoking |
| Carey, K.B. Carey, M.P. Maisto, S.A. Henson, J.M*. “Brief motivational interventions for heavy college drinkers: A randomized controlled trial.”* | 2006 | RCT | USA | Young adults (Stratified by young males) | 18-25 | Alcohol use |
| Caudill, B. D. Luckey, B. Crosse, S. B. Blane, H. I. Ginexi, E. M. Campbell, B. *“Alcohol risk-reduction skills training in a national fraternity: A randomized intervention trial with longitudinal intent-to-treat analysis.”* | 2007 | Cluster RCT | USA | Young adult males only | 18-30 | Alcohol use |
| Cederberg, H. Mikkola, I. Jokelainen, J. Harkonen, P. Ikaheimo, T. Laakso, M. Keinanen-Kiukaanniemi, S. “*Exercise during military training improves cardiovascular risk factors in young men.”* | 2011 | Case series | Finland | Young adult males only | 19-28 | Physical inactivity |
| Cederberg, H. Koivisto, V. M. Jokelainen, J. Surcel, H. M. Keinanen-Kiukaanniemi, S. Rajala, U. *“Unacylated ghrelin is associated with changes in insulin sensitivity and lipid profile during an exercise intervention.”* | 2012 | Case series | Finland | Young adult males only | 19-28 | Physical inactivity |
| Crosby, R. DiClemente, R. J. Charnigo, R. Snow, G. Troutman, A. *“A brief, clinic-based, safer sex intervention for heterosexual African American men newly diagnosed with an STD: a randomized controlled trial.”* | 2009 | RCT | USA | Young adult males only | 18-29 | Unsafe sexual behaviour |
| Daeppen, J. B. Bertholet, N. Gaume, J. Fortini, C. Faouzi, M. Gmel, G*. “Efficacy of brief motivational intervention in reducing binge drinking in young men: A randomized controlled trial.”* | 2011 | RCT | Switzerland | Young adult males only | 20 only | Alcohol use |
| Davidson, D. Swift, R. Fitz, E. “*Naltrexone increases the latency to drink alcohol in social drinkers.”* | 1996 | RCT | USA | Young adults (Stratified by young males) | 21-32 | Alcohol use |
| Denering, L. L. Spear, S. E. *"Routine use of screening and brief intervention for college students in a university counseling center."* | 2012 | Case series | USA | Young adults (Stratified by young males) | 18-24 | Alcohol use, recreational drug use |
| Dermen, Kurt H. Thomas, Sherilyn N. *“Randomized controlled trial of brief interventions to reduce college students' drinking and risky sex”* | 2011 | RCT | USA | Young adults (Stratified by young males) | 18-30 | Alcohol use, sexual behaviour |
| Donnelly, J. E. Hill, J. O. Jacobsen, D. J. Potteiger, J. Sullivan, D. K. Johnson, S. L. Heelan, K. Hise, M. Fennessey, P. V. Sonko, B. Sharp, T. Jakicic, J. M. Blair, S. N. Tran, Z. V. Mayo, M. Gibson, C. Washburn, R. A*. “Effects of a 16-month randomized controlled exercise trial on body weight and composition in young, overweight men and women: the Midwest Exercise Trial”* | 2003 | RCT | USA | Young adults (Stratified by young males) | 17-35 | Physical inactivity |
| Donnelly, J. E. Washburn, R. A. Smith, B. K. Sullivan, D. K. Gibson, C. Honas, J. J. Mayo, M. S. *“A randomized, controlled, supervised, exercise trial in young overweight men and women: the Midwest Exercise Trial II (MET2)”* | 2012 | RCT | USA | Young adults (Stratified by young males) | 18-30 | Physical inactivity |
| Dunn, M. E. Cathy Lau, H. Cruz, I. Y. *“Changes in activation of alcohol expectancies in memory in relation to changes in alcohol use after participation in an expectancy challenge program”* | 2000 | Case series | USA | Young adults (Stratified by young males) | 18-28 | Alcohol use |
| Ergul, S. Temel, A. B. *“The effects of a nursing smoking cessation intervention on military students in Turkey”* | 2009 | Case series | Other (please state) Turkey | Young adult males only | 18-21 | Tobacco smoking |
| Falk, B. Montgomery, H. *“Promoting traffic safety among young male drivers by means of elaboration-based interventions”* | 2009 | Non RCT | Sweden | Young adult males only | 18-23 | Unsafe vehicle driving |
| Fournier, A.K. Ehrhart, I.J. Glindemann, K.E. Geller, E. “Intervening to Decrease Alcohol Abuse at University Parties: Differential Reinforcement of Intoxication Level” | 2004 | Non RCT | USA | Young adults (Stratified by young males) | 19-24 | Alcohol use |
| Garcia, P.J. Holmes, K.K. Carcamo, C.P. Garnett, G.P. Hughes, J.P. Campos, P.E. Whittington, W.L. H. *“Prevention of sexually transmitted infections in urban communities (Peru PREVEN): a multicomponent community-randomised controlled trial.”* | 2012 | Cluster RCT | Peru | Young adults (Stratified by young males) | 18-29 | Unsafe sexual behaviour |
| Gaume, J. Gmel, G. Faouzi, M. Bertholet, N. Daeppen, J. B. *“Is Brief Motivational Intervention Effective in Reducing Alcohol Use Among Young Men Voluntarily Receiving It? A Randomized Controlled Trial”* | 2011 | RCT | Switzerland | Young adult males only | 19 only | Alcohol use |
| Ghahramanloo, E. Midgley, A. W. Bentley, D. J. *“The effect of concurrent training on blood lipid profile and anthropometrical characteristics of previously untrained men”* | 2009 | RCT | Iran | Young adult males only | 23-28 | Physical inactivity |
| Gmel, G. Gaume, J. Bertholet, N. Fluckiger, J. Daeppen, J. B *“Effectiveness of a brief integrative multiple substance use intervention among young men with and without booster sessions”* | 2013 | RCT | Switzerland | Young adult males only | 19 only | Tobacco smoking, alcohol use, recreational drug use |
| Grant, R. M. Lama, J. R. Anderson, P. L. McMahan, V. Liu, A. Y. Vargas, L. Goicochea, P. Casapía, M. Guanira-Carranza, J. V. Ramirez-Cardich, M. E. Montoya-Herrera, O. Fernández, T. Veloso, V. G. Buchbinder, S. P. Chariyalertsak, S. Schechter, M. Bekker, L. G. Mayer, K. H. Kallás, E. G. Amico, K. R. Mulligan, K. Bushman, L. R. Hance, R. J. Ganoza, C. Defechereux, P. Postle, B. Wang, F. McConnell, J. J. Zheng, J. H. Lee, J. Rooney, J. F. Jaffe, H. S. Martinez, A. I. Burns, D. N. Glidden, D. V. *“Preexposure chemoprophylaxis for HIV prevention in men who have sex with men.”* | 2010 | RCT | Brazil, peru, ecuador, USA, Thailand, South Africa. | Adults (Stratified by young males) | 18-25 | Unsafe sexual behaviour |
| Ha, E. J. Caine-Bish, N. Holloman, C. Lowry-Gordon, K.  *“Evaluation of effectiveness of class-based nutrition intervention on changes in soft drink and milk consumption among young adults”* | 2009 | Case series | USA | Young adults (Stratified by young males) | 18-24 | Poor diet |
| Ha, E.J Caine-Bish, N. *“Effect of nutrition intervention using a general nutrition course for promoting fruit and vegetable consumption among college students”* | 2009 | Case series | USA | Young adults (Stratified by young males) | 18-24 | Poor diet |
| Ha, E.J Caine-Bish, N. “Interactive introductory nutrition course focusing on disease prevention increased whole-grain consumption by college students.” | 2011 | Case series | USA | Young adults (Stratified by young males) | 18-24 | Poor diet |
| Hebden, L. Balestracci, K. McGeechan, K. Denney-Wilson, E. Harris, M. Bauman, A. Allman-Farinelli, M. '*TXT2BFiT' a mobile phone-based healthy lifestyle program for preventing unhealthy weight gain in young adults: study protocol for a randomized controlled trial.”* | 2013 | RCT | Australia | Young adults (Stratified by young males) | 18-35 | Poor diet, Physical inactivity |
| Hembroff, L. Atkin, C. Martell, D. McCue, C. Greenamyer, J. T. *“Evaluation Results of a 21st Birthday Card Program Targeting High-Risk Drinking”* | 2007 | RCT | USA | Young adults (Stratified by young males) | 21 only | Alcohol use |
| Hester, R.K. Delaney, H.D. Campbell, W*. ”The College Drinker's Check-Up: Outcomes of two randomized clinical trials of a computer-delivered intervention”* | 2012 | RCT | USA | Young adults (Stratified by young males) | 18-24 | Alcohol use |
| Heydari, M. Boutcher, Y. N. Boutcher, S. H. “High-intensity intermittent exercise and cardiovascular and autonomic function” | 2013 | RCT | Australia | Young adult males only | 18-35 | Physical inactivity |
| Heydari, M. Boutcher, Y. N. Boutcher, S. H. *“The effects of high-intensity intermittent exercise training on cardiovascular response to mental and physical challenge”* | 2013 | RCT | Australia | Young adult males only | 18-35 | Physical inactivity |
| Hightow-Weidman, L. B. Pike, E. Fowler, B. Matthews, D. M. Kibe, J. McCoy, R. Adimora, A. A. *“HealthMpowerment.org: Feasibility and acceptability of delivering an internet intervention to young Black men who have sex with men”* | 2012 | RCT | USA | Young adult males only | 18-30 | Unsafe sexual behaviour |
| Hiruntrakul, A. Nanagara, R. Emasithi, A. Borer, K. T. *“Effect of once a week endurance exercise on fitness status in sedentary subjects”* | 2010 | RCT | Thailand | Young adult males only | 18-25 | Physical inactivity |
| Hosek, S. G. Siberry, G. Bell, M. Lally, M. Kapogiannis, B. Green, K. Fernandez, M. I. Rutledge, B. Martinez, J. Garofalo, R. Wilson, C. M. *“The acceptability and feasibility of an HIV preexposure prophylaxis (PrEP) trial with young men who have sex with men”* | 2013 | RCT | USA | Young adult males only | 18-22 | Unsafe sexual behaviour |
| Howat, P. Robinson, S. Binns, C. Palmer, S. Landauer, A. Educational Biofeedback Driving Simulator as a Drink-Driving Prevention Strategy | 1991 | RCT | Australia | Young adult males only | 18-35 | Unsafe Vehicle driving, alcohol use. |
| Ichiyama, M. A. Fairlie, A. M. Wood, M. D. Turrisi, R. Francis, D. P. Ray, A. E. Stanger, L. A. *“A Randomized Trial of a Parent-Based Intervention on Drinking Behavior Among Incoming College Freshmen”* | 2009 | RCT | USA | Young adults (Stratified by young males) | 18-19 | Alcohol use |
| Kalmuss, D. Armstrong, B. Franks, M. Hecker, G. Gonzalez, J. *“Evaluation of a community-based sexual health intervention for young adult Latino and African-American men”* | 2008 | Non RCT | USA | Young adult males only | 18-30 | Unsafe sexual behaviour |
| Kazemi, D. Dmochowski, J. Sun, L. Grady, K. Nies, MA Walford, S. *“Brief motivational interviewing to reduce alcohol consumption among freshmen: Secondary effects on polydrug use”* | 2012 | Case series | USA | Young adults (Stratified by young males) | 18-20 | Alcohol use, recreational drug use |
| Kazemi, D. Dmochowski, J. Sun, L. Nies, M. Walford, S. *“Outcomes of a targeted capacity expansion (TCE) brief motivational intervention for high-risk drinking freshmen: Pilot study comparison at baseline and 6 months”* | 2011 | Non RCT | USA | Young adults (Stratified by young males) | 18-20 | Alcohol use |
| Kegeles, S.M. Hays, R.B. Coates, T.J. *“The Mpowerment Project: A community-level HIV prevention intervention for young gay men”* | 1996 | Cluster controlled | USA | Young adult males only | 18-29 | Unsafe sexual behaviour |
| Kegeles, S.M. Hays, R.B. Pollack, L.M. Coates, T.J. *“Mobilizing young gay and bisexual men for HIV prevention: A two-community study”* | 1999 | Interrupted time series | USA | Young adult males only | 18-27 | Unsafe sexual behaviour |
| Kennedy, S. B. Nolen, S. Pan, Z. F. Smith, B. Applewhite, J. Vanderhoff, K. J. *“Effectiveness of a brief condom promotion program in reducing risky sexual behaviours among African American men”* | 2013 | RCT | USA | Young adult males only | 18-24 | Unsafe sexual behaviour |
| Kirk, E. P. Washburn, R. A. Bailey, B. W. LeCheminant, J. D. Donnelly, J. E. *“Six months of supervised high-intensity low-volume resistance training improves strength independent of changes in muscle mass in young overweight men”* | 2007 | RCT | USA | Young adult males only | 18-25 | Physical inactivity |
| Kypri, K. McCambridge, J. Vater, T. Bowe, S. J. Saunders, J. B. Cunningham, J. A. Horton, N. J. *“Web-based alcohol intervention for Maori university students: double-blind, multi-site randomized controlled trial* | 2012 | RCT | New Zealand | Young adults (Stratified by young males) | 17-24 | Alcohol use |
| Labrie, J. Migliuri, S. Cail, J. *“A night to remember: A harm-reduction birthday card intervention reduces high-risk drinking during 21st birthday celebration”* | 2009 | Cluster RCT | USA | Young adults (Stratified by young males) | 21 only | Alcohol use |
| Lee, C. M. Neighbors, C. Kilmer, J. R. Larimer, M. E. “*A brief, web-based personalized feedback selective intervention for college student marijuana use: a randomized clinical trial”* | 2010 | RCT | USA | Young adults (Stratified by young males) | 17-19 | Recreational drug use |
| Lenne, M.G. Liu, C.C. Salmon, P.M. Holden, M. Moss, S. *“Minimising risks and distractions for young drivers and their passengers: An evaluation of a novel driver-passenger training program”* | 2011 | RCT | Australia | Young adult males only | 18-21 | Unsafe vehicle driving |
| Lewis, M.A. Neighbors, C. Lee, C.M. Oster-Aaland, L. *“21st birthday celebratory drinking: Evaluation of a personalized normative feedback card intervention”* | 2008 | RCT | USA | Young adults (Stratified by young males) | 21 only | Alcohol use |
| Marlatt, G. Baer, J.S. Kivlahan, D.R. Dimeff, L.A. Larimer, M.E. Quigley, L.A. Somers, J.M. Williams, E. *“Screening and brief intervention for high-risk college student drinkers: Results from a 2-year follow-up assessment”* | 1998 | RCT | USA | Young adults (Stratified by young males) | 17-19 | Alcohol use |
| Marra, C. Bottaro, M. Oliveira, R. J. Novaes, J. S. *“Effect of moderate and high intensity aerobic exercise on the body composition of overweight men”* | 2005 | RCT | Brazil | Young adult males only | 18-33 | Physical inactivity |
| Milhausen, R. R. Wood, J. Sanders, S. A. Crosby, R. A. Yarber, W. L. Graham, C. A*. “A novel, self-guided, home-based intervention to promote condom use among young men: a pilot study”* | 2011 | Case series | Canada | Young adult males only | 18-21 | Unsafe sexual behaviour |
| Moyer-Gusé, E. Nabi, R. L. *“Comparing the effects of entertainment and educational television programming on risky sexual behaviour”* | 2011 | RCT | USA | Young adults (Stratified by young males) | 18-25 | Unsafe sexual behaviour |
| Murphy TJ, Pagano RR, Marlatt GA. Lifestyle modification with heavy alcohol drinkers: effects of aerobic exercise and meditation. | 1986 | RCT | USA | Young adult males only | 21-30 | Alcohol use, physical inactivity |
| Neighbors, C. Lee, C.M. Atkins, D.C. Lewis, M.A. Kaysen, D. Mittmann, A. Fossos, N. Geisner, I.M. Zheng, C. Larimer, M.E. *“A randomized controlled trial of event-specific prevention strategies for reducing problematic drinking associated with 21st birthday celebrations”* | 2012 | RCT | USA | Young adults (Stratified by young males) | 21 only | Alcohol use |
| Ozdemir, R. A. Celik, O. Asci, F. H. *“Exercise interventions and their effects on physical self-perceptions of male university students”* | 2010 | RCT | Turkey | Young adult males only | 19-25 | Physical inactivity |
| Prokhorov, A. V. Yost, T. Mullin-Jones, M. de Moor, C. Ford, K. H. Marani, S. Kilfoy, B. A. Hein, J. P. Hudmon, K. S. Emmons, K. M. *"Look At Your Health": Outcomes associated with a computer-assisted smoking cessation counseling intervention for community college students”* | 2008 | Cluster RCT | USA | Young adults (Stratified by young males) | 18-35 | Tobacco smoking |
| Raz, I. Israeli, A. Rosenblit, H. Bar-On, H*. “Influence of moderate exercise on glucose homeostasis and serum testosterone in young men with low HDL-cholesterol level”* | 1988 | RCT | Isreal | Young adult males only | 24-26 | Physical inactivity |
| Raz, I. Rosenblit, H. Kark, J. D. *“Effect of moderate exercise on serum lipids in young men with low high density lipoprotein cholesterol”* | 1988 | RCT | Isreal | Young adult males only | 24-26 | Physical inactivity |
| Rivara, FP. Boisvert, D. Relyea-Chew, A. Gomez, T. *“Last Call: decreasing drunk driving among 21-34-year-old bar patrons”* | 2012 | Interrupted time series | USA | Young adults (Stratified by young males) | 21-34 | Unsafe vehicle driving |
| Roberts, C. K. Croymans, D. M. Aziz, N. Butch, A. W. Lee, C. C. *“Resistance training increases SHBG in overweight/obese, young men”* | 2013 | RCT | USA | Young adult males only | 18-35 | Physical inactivity |
| Rohsenow DJ, Smith RE, Johnson S. Stress management training as a prevention program for heavy social drinkers: cognitions, affect, drinking, and individual differences. | 1985 | RCT | USA | Young adult males only | 20-24 | Alcohol use |
| Rossomanno, C.I. Herrick, J.E. Kirk, S.M. Kirk, E.P. “*A 6-month supervised employer-based minimal exercise program for police officers improves fitness”* | 2012 | Case series | USA | Young adults (Stratified by young males) | 21-35 | Physical inactivity |
| Sallis, J. F. Calfas, K. J. Nichols, J. F. Sarkin, J. A. Johnson, M. F. Caparosa, S. Thompson, S. Alcaraz, J. E. “*Evaluation of a university course to promote physical activity: Project GRAD”* | 1999 | RCT | USA | Young adults (Stratified by young males) | 18-29 | Physical inactivity |
| Sanderson, C. A. *“Role of relationship context in influencing college students' responsiveness to HIV prevention videos”* | 1999 | RCT | USA | Young adults (Stratified by young males) | 17-24 | Unsafe sexual behaviour |
| Sandström, B. Marckmann, P. Bindslev, N. *“An eight-month controlled study of a low-fat high-fibre diet: effects on blood lipids and blood pressure in healthy young subjects”* | 1992 | Non RCT | Denmark | Young adults (Stratified by young males) | 20-30 | Poor diet |
| Shaw, I. Shaw, B. S. “*Relationship between resistance training and lipoprotein profiles in sedentary male smokers.”* | 2008 | Case series | South Africa | Young adult males only | 20-35 | Physical inactivity |
| Shepherd, J. Weare, K. Turner, G. Peer-led sexual health promotion with young gay and bisexual men - results of The HAPEER Project | 1997 | Non RCT | UK | Young adult males only | 17-25 | Unsafe sexual behaviour |
| Shorey, R. L. Sewell, B. O'Brien, M. *“Efficacy of diet and exercise in the reduction of serum cholesterol and triglyceride in free-living adult males”* | 1976 | Non RCT | USA | Young adult males only | 22-30 | Poor diet, Physical inactivity |
| Simmons, V. N. Brandon, T. H. *“Secondary smoking prevention in a university setting: A randomized comparison of an experiential, theory-based intervention and a standard didactic intervention for increasing cessation motivation”* | 2007 | RCT | USA | Young adults (Stratified by young males) | 18-24 | Tobacco smoking |
| Somerville, G.G. Diaz, S. Davis, S Coleman, K.D. Taveras, S *“Adapting the Popular Opinion Leader Intervention for Latino Young Migrant Men Who Have Sex With Men”* | 2006 | Case series | USA | Young adult males only | 18-30 | Unsafe sexual behaviour |
| Spalding, T. W. Lyon, L. A. Steel, D. H. Hatfield, B. D *“Aerobic exercise training and cardiovascular reactivity to psychological stress in sedentary young normotensive men and women”* | 2002 | RCT | USA | Young adults (Stratified by young males) | 19-29 | Physical inactivity |
| Taubman-Ben-Ari, O. Lotan, T. *“The contribution of a novel intervention to enhance safe driving among young drivers in Israel”* | 2011 | Non RCT | Israel | Young adults (Stratified by young males) | 17-21 | Unsafe vehicle driving |
| Tyden, T. Bergholm, M. Hallen, A. Odlind, V. Olsson, S. Sjoden, P. Strand, A. Bjorkelund, C. *“Evaluation of an STD-prevention program for Swedish university students”* | 1998 | Non RCT | Sweden | Young adults (Stratified by young males) | 18-25 | Unsafe sexual behaviour |
| Ulla Diez, S.M. Fortis, A.P. Franco, S.F. “*Efficacy of a health-promotion intervention for college students”* | 2012 | RCT | Mexico | Young adults (Stratified by young males) | 17-24 | Poor diet, Physical inactivity |
| Voogt, C. V. Poelen, E. A. P. Kleinjan, M. Lemmers, L. Engels, R. *“Targeting young drinkers online: the effectiveness of a web-based brief alcohol intervention in reducing heavy drinking among college students: study protocol of a two-arm parallel group randomized controlled trial”* | 2011 | RCT | Netherlands | Young adults (Stratified by young males) | 18-24 | Alcohol use |
| Walsh, L.A. Stock, M.L. *“UV photography, masculinity, and college men's sun protection cognitions”* | 2012 | Case series | USA | Young adult males only | 18-22 | Tanning/sun exposure |
| Washburn, R. A. Donnelly, J. E. Smith, B. K. Sullivan, D. K. Marquis, J. Herrmann, S. D. *“Resistance training volume, energy balance and weight management: rationale and design of a 9 month trial”* | 2012 | RCT | USA | Young adults (Stratified by young males) | 18-30 | Physical inactivity |
| Weisse, C. S. Turbiasz, A. A. Whitney, D. J. *“Behavioral training and aids risk reduction: Overcoming barriers to condom use”* | 1995 | RCT | USA | Young adult males only | 17-23 | Unsafe sexual behaviour |
| White, M. J. Cunningham, L. C. Titchener, K. *“Young drivers' optimism bias for accident risk and driving skill: Accountability and insight experience manipulations”* | 2011 | RCT | Australia | Young adults (Stratified by young males) | 17-25 | Unsafe vehicle driving |
| Williams, D. R. Lewis, N. M. *“Effectiveness of nutrition counseling in young adult males”* | 2002 | RCT | USA | Young adult males only | 20-25 | Poor diet |
| Wolitski, R.J. *“Relative Efficacy of a Multisession Sexual Risk-Reduction Intervention for Young Men Released from Prisons in 4 States”* | 2006 | Cluster controlled | USA | Young adult males only | 18-29 | Unsafe sexual behaviour |
| Wood, M.D. Capone, C. Laforge, R. Erickson, D.J. Brand, N.H. *“Brief motivational intervention and alcohol expectancy challenge with heavy drinking college students: A randomized factorial study”* | 2007 | RCT | USA | Young adults (Stratified by young males) | 20-24 | Alcohol use |
| Wood, M.D. Fairlie, A.M. Fernandez, A.C. Borsari, B. Capone, C. Laforge, R. Carmona-Barros, R *“Brief motivational and parent interventions for college students: A randomized factorial study”.* | 2010 | RCT | USA | Young adults (Stratified by young males) | 17-21 | Alcohol use |
| Wu, Z. Detels, R. Zhang, J. Li, V. Li, J. *“Community-based trial to prevent drug use among youths in Yunnan, China”* | 2002 | Cluster controlled | China | Adult Males (Stratified by young males) | 20-29 | Recreational drug use |
