# Supplementary figures and images for: A scoping review of risk behaviour interventions in young men
Source: BMC Public Health. 2014 Sep 16;14:957. doi: 10.1186/1471-2458-14-957 (PMC4177699; doi:10.1186/1471-2458-14-957)

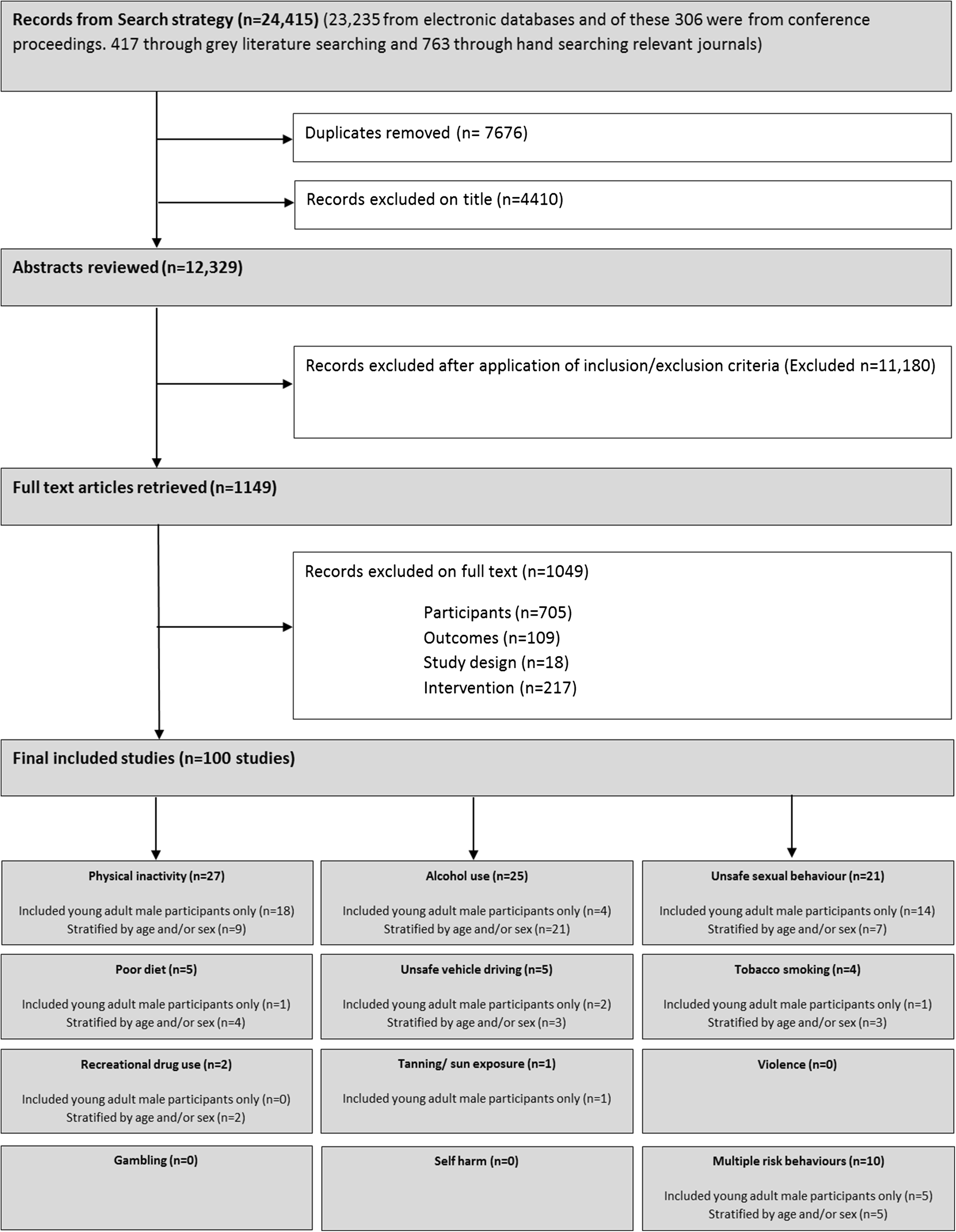

Supplement: Supplementary file 3 — Authors’ original file for figure 1 [file 12889_2014_7077_MOESM3_ESM.tif]
